# A latent class analysis approach to the identification of doctoral students at risk of attrition

**DOI:** 10.1371/journal.pone.0280325

**Published:** 2023-01-13

**Authors:** Samantha M. Stevens, Peter M. Ruberton, Joshua M. Smyth, Geoffrey L. Cohen, Valerie Purdie Greenaway, Jonathan E. Cook

**Affiliations:** 1 Department of Psychology, The Pennsylvania State University, University Park, PA, United States of America; 2 Department of Biobehavioral Health, The Pennsylvania State University, University Park, PA, United States of America; 3 Department of Psychology and Graduate School of Education, Stanford University, Stanford, CA, United States of America; 4 Department of Psychology, Columbia University, New York, NY, United States of America; Public Library of Science, UNITED STATES

## Abstract

To advance understanding of doctoral student experiences and the high attrition rates among Science, Technology, Engineering, and Mathematics (STEM) doctoral students, we developed and examined the psychological profiles of different types of doctoral students. We used latent class analysis on self-reported psychological data relevant to psychological threat from 1,081 incoming doctoral students across three universities and found that the best-fitting model delineated four threat classes: *Lowest Threat*, *Nonchalant*, *Engaged/Worried*, and *Highest Threat*. These classes were associated with characteristics measured at the beginning of students’ first semester of graduate school that may influence attrition risk, including differences in academic preparation (e.g., amount of research experience), self-evaluations and perceived fit (e.g., sense of belonging), attitudes towards graduate school and academia (e.g., strength of motivation), and interpersonal relations (e.g., perceived social support). *Lowest Threat* students tended to report the most positive characteristics and *Highest Threat* students the most negative characteristics, whereas the results for *Nonchalant* and *Engaged/Worried* students were more mixed. Ultimately, we suggest that *Engaged/Worried* and *Highest Threat* students are at relatively high risk of attrition. Moreover, the demographic distributions of profiles differed, with members of groups more likely to face social identity threat (e.g., women) being overrepresented in a higher threat profile (i.e., *Engaged/Worried* students) and underrepresented in lower threat profiles (i.e., *Lowest Threat* and *Nonchalant* students). We conclude that doctoral students meaningfully vary in their psychological threat at the beginning of graduate study and suggest that these differences may portend divergent outcomes.

## Introduction

Nearly half the doctoral students in Science, Technology, Engineering, and Mathematics (STEM) do not graduate [1]. Although students leave for a variety of reasons (e.g., financial barriers, life circumstances), their psychological experiences, particularly experiences of psychological threat (e.g., stress, belonging concerns, self-doubt), may be a potent explanation for attrition that has been understudied. Aversive psychological experiences may lead directly to students terminating their graduate career or indirectly by undermining motivation and performance. Given that Ph.D. students are poised to contribute significantly to advancing knowledge as the next generation of scholars and innovators, as well as uncertainty in the literature about why so many students attrit, more research is needed on Ph.D. students’ psychological experiences and identifying the types of students whose psychological experiences may predict greater attrition risk. To address this gap, we use latent class analysis to identify psychological profiles of STEM Ph.D. students at matriculation. Our goal is to describe how Ph.D. students vary by psychological threat using an array of characteristics and psychological experiences and expectations at the start of graduate school that may reflect attrition risk.

### Psychological threat

Most of the extant research on Ph.D. student attrition focuses on structural factors (e.g., inadequate financial support) to explain attrition [2]. However, accounting for structural factors, there remains substantial unexplained variance in Ph.D. student attrition [2–4], suggesting a need for other explanations [1]. A body of literature at pre-doctoral levels suggests psychological experiences can greatly affect student motivation, and we postulate that these experiences may also matter for Ph.D. students. We consider psychological experiences under the rubric of psychological threat, which we define as the psychological state that emerges when a situation poses risks to one’s sense of global self-integrity [5].

There are many reasons to suspect that Ph.D. students, across fields and identity groups, contend with psychological threat. Ph.D. students face a barrage of novel challenges with few clear milestones and relatively little assurance of success. Ph.D. students likely experience more failure, rejection, and critical feedback than they have before alongside new professional and social norms of a scholarly community that can be psychologically exhausting [6]. Even before they begin their studies, Ph.D. students may worry about their ability to succeed, which can impair performance and motivation to persist [7]. Additionally, Ph.D. students might feel like frauds [8] while also contending with ongoing uncertainty, such as whether they will attain long-term goals [9].

Many constructs related to psychological threat identified as influential for academic outcomes at lower levels of education are likely relevant for Ph.D. students. Such constructs include growth mindset [10], grit [11], academic identification [12], self-efficacy [13], and sense of belonging [14, 15]. These constructs draw from various theories in social and educational psychology, including the expectancy-value theory of achievement motivation [7], the biopsychosocial model of challenge and threat [16], and social identity threat [17].

It seems likely that Ph.D. students vary substantially in at least some of these constructs, and this variation could be tied to different outcomes (e.g., attrition vs retention). Together, threatening experiences and tenuous psychological states among Ph.D. students could create a particularly threatening psychological climate, one that would be difficult to manage and that would help explain high attrition rates. However, there is little research on the psychological experiences of Ph.D. students, particularly as they relate to attrition, which is notable given Ph.D. students, particularly those in STEM, are poised to become leaders in research and innovation.

Social identity threat (SIT), a concern that one will be devalued due to one or more social group memberships [17], may help explain why attrition rates [1] and worse academic outcomes in doctoral education, like lower publication rates [18, 19], are higher for women and members of underrepresented racial/ethnic minority (URM) groups in STEM fields. Negative stereotypes about the intelligence of women and URM members are well-known, and, along with other environmental characteristics, can generate the conditions where SIT becomes salient and contributes to achievement gaps over time [20–25]. We suggest that the disproportionate attrition rates for these groups in doctoral education may be partially explained by SIT.

SIT is an additional form of psychological threat on top of the psychological threats other students may face. For example, it is likely normative among Ph.D. students to sometimes worry about being seen as incompetent (general threat). However, students who worry that others are negatively evaluating them due to their race or gender (SIT) carry an additional psychological burden that may help explain their greater attrition. In addition to negative stereotypes, one cue that amplifies SIT is perceiving oneself to be a numerical minority, relevant in doctoral education given the underrepresentation of certain groups in many STEM fields. SIT is also heightened when people are highly identified with a domain and want to succeed [26], both highly likely for Ph.D. students.

Doctoral students may thus contend with an array of psychological threats, both at the beginning of graduate school and throughout their studies. Importantly, some students may face general psychological threats *and* SIT, while others face one or none. If psychological threat in general, and SIT in particular, are risk factors in doctoral education, it would help to identify students who, based on their threat pattern at the start of their studies, may benefit from early intervention. To work toward this goal, we use latent class analysis to identify student profiles of psychological threat at the start of doctoral education.

### Latent class analysis

As noted, many variables related to psychological threat and SIT help explain achievement gaps at pre-doctoral levels of education. One approach to identifying Ph.D. students at risk of attrition is to regress attrition or other relevant outcomes on many or all of these potentially influential variables, plus their products, to account for specific combinations that may best predict educational outcomes. However, this kind of variable-centered approach requires many predictors, making models unwieldy and introducing multicollinearity. Indeed, variable-centered approaches often cannot examine complex higher-order interactions due to issues like statistical power [27] and thus obscure meaningful patterns among individuals. Mixture models, like latent class analysis (LCA), offer an alternative.

Mixture models are person-centered and reveal subgroups of people who share similar responses on a set of observed variables [28]. LCA is a type of mixture model used when one suspects that an unobserved categorical variable separates a population into mutually exclusive and exhaustive subgroups, or latent classes [29]. LCA can be thought of as a data reduction tool, as it distills a great deal of information into identifiable patterns reflected in the sample. A regression analysis analogue to an LCA with eight 3-level categorical indicators (i.e., observed variables used to differentiate classes) would require 3^8^ = 6,561 possible subgroups to examine every possible pattern of responses. With LCA, we can reduce these subgroups to a few meaningful ones. LCA can parsimoniously show how academic risk factors interact, including how prevalent different risks (and subgroups) are.

Moreover, we can explore how identified subgroups differ on constructs aside from the indicators by examining associations with proximal outcomes, or characteristics. For instance, we can examine if subgroups characterized by ostensibly more threatening patterns of traits feel more negatively about graduate school than lower threat subgroups. By using LCA in this way, we take a critical first step towards long-term goals of determining where interventions should be applied by identifying where students fall along a threat continuum [30].

### Overview of current research

In the present research, we use data from the *Study for the Advancement of Graduate Education and Scholarship* (SAGES) to better understand the psychological experiences of Ph.D. students at the start of graduate school. SAGES is a prospective multisite study of the psychological experiences of Ph.D. students that predict attrition and retention, particularly in STEM fields. We conducted an unrestricted LCA to delineate threat profiles (AKA classes) using data from a baseline survey completed by incoming Ph.D. students. Then, we examined how the selected model mapped onto student demographics, expecting differences consistent with past literature. For instance, female Ph.D. students in many STEM fields may experience identity threat and thus, be overrepresented in high SIT profiles. To better understand the profiles, we also examined differences between them on proximal indicators of threat important for academic success. Our goal was to identify profiles of incoming Ph.D. students with an eye towards understanding how these may have different risks for negative outcomes like attrition.

## Method

The overall design and hypotheses of SAGES were pre-registered (https://bit.ly/3hNrjPL) including the hypothesis that women, first-generation, and URM students will have higher levels of psychological threat at the start of graduate school and a higher risk of attrition. We also outlined the use of LCA to create a composite threat variable based on past literature. Materials, data, and code are available at https://bit.ly/3hNrjPL.

### Participants

Participants were two cohorts of first-semester Ph.D. students at three universities (Penn State, Columbia, and Stanford) who completed a baseline survey in Fall 2018 or 2019. All first-year STEM Ph.D. students were eligible to participate, and a smaller number of non-STEM students were targeted at Penn State. Results are based on the 1,081 students who completed the baseline survey (see Table 1). The sample of 1,081 includes three students with missing responses on one or more indicators because LCA uses maximum likelihood estimation, which allows partial data on indicators. However, this sample excludes 44 students who did not complete the baseline survey (i.e., who stopped responding before the end) but still provided data on LCA indicators. We conducted the final unrestricted 4-class LCA including these students (total *N* = 1,125) and found the same latent classes and a similar percentage of people in each class (see S1 Appendix).

**Table 1:** Demographic information.

	*n* (proportion)	Mean *(SD)*	Range
**Age**	1069	24.17 (3.36)	19–55
**Socioeconomic status**	1074	6.07 (1.80)	1–10
**Gender**			
Genderqueer	11 (.01)		
Female	522 (.48)		
Female	519 (.48)		
Female and Genderqueer	3 (.003)		
Male	548 (.51)		
Male	544 (.50)		
Male and Genderqueer	1 (.0009)		
Male and Trans Male and Genderqueer	1 (.0009)		
Trans Male and Genderqueer	1 (.0009)		
Trans Male	1 (.0009)		
**Race/ethnicity**			
Asian	122 (.113)		
Black	24 (.022)		
Hispanic	27 (.025)		
Multiracial	40 (.037)		
Asian/White	13 (.012)		
Other multiracial identity	27 (.025)		
Native American	2 (.002)		
White	406 (.376)		
American, race unknown	3 (.003)		
International	457 (.423)		
**Sexual orientation**			
Asexual	16 (.015)		
Bisexual	111 (.103)		
Gay/Lesbian	56 (.052)		
Straight	864 (.803)		
Other	29 (.027)		
**First-generation status**			
Yes	218 (.203)		
No	857 (.797)		
**Region of birth for international students**			
Africa	12 (.026)		
Arab States	3 (.007)		
Asia & Pacific	324 (.709)		
Europe	53 (.116)		
Middle east	29 (.063)		
North America	9 (.020)		
South/Latin America	26 (.057)		

The maximum *n* is 1081 (number of students used in final analytic sample for unrestricted LCA).

Participants’ age ranged from 19 to 55 *(M* = 24.17) and their gender was nearly evenly female (522) and male (548), with 11 identifying as genderqueer. Where data were available, we found that this sample was reasonably representative of the population, with the most notable difference being that women were overrepresented in our Cohort 1 Stanford sample (see S2 Appendix for details).

### Procedures

Written Institutional Review Board (IRB) approval for SAGES was obtained at all sites (STUDY00007231, IRB-AAAR3748, and 28910 for Penn State, Columbia, and Stanford, respectively). We primarily recruited students by email but also attended in-person orientation sessions for new Ph.D. students. Recruitment details and timing varied slightly across campuses (see S3 Appendix), but generally started two weeks before the school year’s start and concluded two weeks after classes began. We described the study as about understanding Ph.D. student experiences. Recruitment materials included a link to a baseline survey to assess students’ thoughts, behaviors, and characteristics before starting graduate school. Participants who completed the 45-minute baseline survey were paid $15 and invited to participate in the longitudinal part of SAGES (not reported here). Students could participate in the baseline survey and not the longitudinal study.

The recruitment procedure was similar across campuses. Most students were recruited with help from university administrators, who sent a recruitment email written by the research team to all incoming Ph.D. students in identified STEM fields (see https://bit.ly/3hNrjPL for a list), and a small number of non-STEM fields at Penn State. The email included an introduction from each dean that noted support for the study but assured students that their participation would not be known by the university or affect their graduate career. At Stanford, we did not have a way to email incoming STEM Ph.D. students in one college, so we sent the recruitment email (without a dean’s message) to departmental administrators in that college with a request to forward the message to incoming Ph.D. students. We also attended in-person orientations for Ph.D. students at Stanford and Penn State where we handed out fliers. Students outside of the targeted fields could participate if they became aware of the study in this way. We did not have information about non-participating students, but the Graduate School at Penn State provided limited demographic data for all incoming students by field.

### Measures

To develop a comprehensive set of measures, we spent several months identifying constructs relevant to psychological threat and persistence in academic settings and conducted an exhaustive literature review to identify the most relevant and psychometrically sound scales. We often selected measures used in research about lower levels of education given scarcity of research on doctoral education. We consulted an advisory board convened for this project and other experts in psychology, education, and STEM graduate education and disparities. We pilot tested measures with Ph.D. students to ensure clarity and face validity. To capture a comprehensive set of constructs relevant to psychological threat and attrition and in line with other longitudinal studies [31], we shortened scales where possible, basing decisions on psychometric properties (e.g., dropping items with lowest factor loadings) and face validity. We also changed response scales in some cases to be consistent with other measures.

Below, we list measures used as indicators for the LCA and as proximal outcomes to further characterize and validate the observed classes. We start with demographics and then describe measures by their placement in one of six superordinate categories we created to promote organization: academic preparation and context, self-evaluations and perceived fit, academic identity and graduate school attitudes, interpersonal relations, mental health, and SIT. As described below, we selected measures based on their association with psychological threat and/or attrition risk. We examined threat-relevant measures not used as indicators as proximal characteristics to enrich understanding of each class’s psychological state (see Analytic Strategy). Unless otherwise noted, we averaged scale items; higher scores indicate more of the construct. See Table 2 for descriptive statistics.

**Table 2:** Descriptive statistics on proximal characteristics.

Variable	*n*	Mean (*SD)*	Range
**Academic Preparation & Context**			
Proportion of women in field	1080	0.38 (0.17)	0.13–0.73
Years undergrad research	1077	1.87 (1.09)	0–6
Years postgrad research	1074	1.08 (1.63)	0–20
Undergrad research preparation	996	3.16 (1.06)	1–5
Postgrad research preparation	545	3.74 (1.02)	1–5
English proficiency	438	4.48 (1.02)	2–6
**Self-Evaluations and Perceived Fit**			
Neuroticism	1081	4.09 (1.36)	1–7
Self-esteem	1080	3.17 (1.18)	1–5
Self-efficacy	1080	3.08 (0.62)	1–4
Academic and social concerns	1080	4.20 (1.24)	1–7
Grit	1081	3.48 (0.66)	1.12–5
Psychological need satisfaction	1081	4.94 (0.77)	1.83–7
Need fulfillment composite	1080	0.00 (0.83)	-3.66–1.96
Academic belonging	1081	5.14 (0.79)	2–7
Belonging uncertainty	1080	4.43 (1.43)	1–7
**Academic Identity & Graduate School Attitudes**			
Interest composite	1081	6.18 (0.77)	2–7
Researcher identification	1081	5.31 (0.95)	1.60–7
Decision Confidence	1081	3.13 (0.62)	1.50–4
Strength of motivation	1081	4.88 (1.00)	1.50–7
Academic self-control	1081	3.17 (0.91)	1–5
Impostor syndrome	1080	3.20 (0.93)	1–5
Academic career preference	1078	1.46 (2.88)	-5–5
**Interpersonal Relations**			
Perceived social support	1079	4.03 (0.90)	1–5
Similarity to colleagues	1079	3.77 (1.33)	1–6
**Mental Health**			
Distress	1079	7.04 (4.26)	0–24
**Social Identity Threat**			
Stereotype threat-race	1079	2.65 (1.43)	1–7
Stereotype threat- gender	1079	2.69 (1.36)	1–7
Gender threat composite	1080	2.68 (1.42)	1–7
Identity interference	1080	2.73 (1.46)	1–7
	*N* (proportion) yes	*N* (proportion) no	
Has master’s	316 (.292)	765 (.708)	

### Demographic characteristics

Table 1 provides demographic characteristics. Participants reported gender identity by selecting one or more of female, male, trans female, trans male, genderqueer or non-conforming, or entering a response [32]. For sexual orientation, participants selected among heterosexual or straight, gay or lesbian, asexual, bisexual, or entered a response. For race/ethnicity, participants self-reported in an open-text response and, on another page, selected any of several categories that applied. For first-generation status, participants reported whether they considered themselves a first-generation college student. For socioeconomic status (SES), using one of the MacArthur Scales of Subjective Social Status, participants selected a rung on a ladder from 1 to 10 to represent where they currently stood relative to others in the United States [33]. We asked students their country of birth, citizenship status, and for U.S. citizens or permanent residents not born in the U.S., the age at which they entered. We used this information to categorize participants as international or domestic (see S4 Appendix for details).

#### Academic preparation and context

For academic preparation, we examined students’ previous research and educational experiences, which have been shown to be associated with persistence in doctoral education [34], and how well students felt prepared for graduate school, as high preparedness, particularly among women and minority Ph.D. students, has been linked to higher rates of publishing [18]. For context, we examined the percentage of women in each field, which can speak to the potential for gender-based SIT, and English proficiency, which can speak to the potential for threat based on a language barrier.

*Percentage women in field*. We approximated the percentage of women in each field using the NSF Survey of Earned Doctorates [35]. See S5 Appendix for details. This contextualizing information sheds light on whether women are underrepresented in a given field, a contributor to gender-based SIT.

*Years undergrad/postgrad research*. Students reported the number of years of research experience they had during and after college.

*Undergrad/postgrad research prep*. Author-generated items assessed how well students felt that their previous research experience during and after college (if applicable) had prepared them for doctoral education, from 1 (*not well at all*) to 5 (*extremely well*). Research suggests that feeling unprepared for doctoral education is a contributor to attrition [36].

*Has master’s*. One dichotomous (yes/no) item assessed whether participants had received a terminal master’s degree prior to starting their doctoral program.

*English proficiency*. Students who reported being non-native in English were asked how well they spoke English from 1 (*Little or no English*: *No proficiency)* to 6 (*Complete fluency*).

#### Self-evaluations and perceived fit

Neuroticism, self-esteem, and self-efficacy are theorized to share the same underlying construct, which can be called *core self-evaluations* [37]. We were interested in these measures as well as academic and social concerns, grit, psychological need satisfaction, academic belonging, and belonging uncertainty as other forms of self-evaluation and perceived fit. More positive self-evaluations are linked to greater task persistence and performance [38, 39].

*Neuroticism*. Three neuroticism items were taken from John et al.’s [40] measure of Big Five personality (e.g., “I am someone who worries a lot”) and rated from 1 (*strongly disagree*) to 7 (*strongly agree*) (α = .76). Higher levels of neuroticism predict worse mental and physical health outcomes [41].

*Self-esteem*. A well-established single-item scale assessed self-esteem, “I have high self-esteem” [42]. Responses ranged from 1 (*not at all true*) to 5 (*very true*).

*Graduate school self-efficacy*. Three items adapted from Shryock and Froyd’s [43] 8-item engineering self-efficacy scale assessed graduate school self-efficacy (e.g., “I expect to do well in graduate school”). Students responded from 1 (*not at all true of me*) to 4 (*very true of me*) (α = .79). Higher self-efficacy generally predicts better college performance [13].

*Academic and social concerns*. Four items, adapted from Cohen and Garcia [44] to reflect a graduate school context, tap academic and social concerns, or worries, about being negatively evaluated (e.g., “I worry that people in my graduate program will think I’m dumb if I do badly”). Participants responded from 1 (*strongly disagree*) to 7 (*strongly agree*) (α = .77). High levels of worry, measured in various ways, can worsen performance [45, 46].

*Grit*. Eight items adapted from Duckworth and Quinn [47] assessed grit (e.g., “I finish whatever I begin”) on a scale from 1 (*not like me at all*) to 5 (*very much like me*) (α = .77). Higher levels of grit have predicted higher grades, a greater sense of belonging, and more college satisfaction among undergraduates [11].

*Psychological need satisfaction*. This scale includes three subscales based on self-determination theory: the need for competence (e.g., “I successfully complete difficult tasks and projects”), relatedness (e.g., “I feel close and connected with other people who are important to me”), and autonomy (e.g., “I am free to do things my own way”). Students responded from 1 (*not at all true*) to 7 (*extremely true*). The 18 items, 6 for each subscale, were adapted from Sheldon and Hilpert [48] to measure current, rather than past, psychological experiences. To limit survey length, we removed two items from each subscale for the second cohort, so we averaged only the 12 items completed in both cohorts (α = .78). Greater need satisfaction, as measured by similar scales, predicts greater academic engagement [49].

*Academic belonging*. We adapted the nine items from the Social and Academic Fit Scale [24] used by Cook et al. [21], to reflect a graduate context (α = .81). Five items assessed social belonging (e.g., “People in my program accept me”) and four items assessed potential to succeed (e.g., “I know what I need to do to succeed in grad school”). Students responded from 1 (*strongly disagree*) to 7 (*strongly agree*). Greater sense of belonging, measured in various ways, is linked to better academic outcomes [14].

*Belonging uncertainty*. Three items adapted from Walton and Cohen [24] captured belonging uncertainty from 1 (*strongly disagree*) to 7 (*strongly agree*): “Sometimes I feel like I belong in grad school and sometimes I feel like I don’t belong,” “When something good happens, I feel like I really belong in grad school,” and “When something bad happens, I feel like maybe I don’t belong in grad school.” Students answered based on their experiences in graduate school so far. Reliability was somewhat low (α = .61), but items 1 and 3 were adequately correlated (*r* = .62), so we present results for the average of these in the main text and for the individual items in the S6 Appendix. Greater uncertainty about belonging undermines academic motivation and performance for stigmatized group members [24].

#### Academic identity and graduate school attitudes

This category includes interest, academic identification, confidence in the decision to pursue a Ph.D., strength of motivation to finish the Ph.D., academic self-control, impostor syndrome, and preference for an academic career. Academic identity and graduate school attitudes can predict academic persistence [50].

*Interest*. We assessed interest in research (e.g., “I am interested in my research topic”) and field (e.g., “I am interested in learning more about my field of study”) with three of four items from Choe et al. [51], rephrased to apply across fields. Participants responded from 1 (*strongly disagree*) to 7 (*strongly agree*) (α = .84). Greater interest, measured similarly, predicts retention in undergraduate STEM fields [52].

*Researcher identification*. We assessed identification as a researcher using three items adapted from Sellers and colleagues’ centrality subscale [53] and two items from Choe et al. [51]. Participants responded from 1 (*strongly disagree*) to 7 (*strongly agree*) (α = .80). Higher levels of identification with one’s studies, measured in various ways, are associated with greater academic persistence at the undergraduate level [54].

*Decision confidence*. Students responded to two author-generated items on how often they felt they made the right choice in pursuing a Ph.D. from 1 (*never*) to 4 (*always*) and if they ever doubted this decision from 1 (*I never doubt my decision*) to 4 (*I frequently doubt my decision*). We reverse-coded the latter item and averaged the two given their adequate correlation (*r* = .65).

*Strength of motivation*. Four items, taken from a 16-item scale [55] and reworded for a graduate school context, captured strength of motivation to continue the Ph.D. (e.g., “Even if I could hardly maintain my social life, I would still continue graduate school”). Students responded from 1 (*strongly disagree*) to 7 (*strongly agree*). Reliability was low (α = .55), but analysis of individual items yields the same pattern (see S6 Appendix).

*Academic self-control*. Academic self-control was measured with two of four items from Yeager et al. [56] (e.g., “I pay attention and resist distraction in my work”), rephrased for a graduate student population. Participants responded from 1 (*not at all like me*) to 5 (*very much like me*). We averaged the items because they were adequately correlated (*r* = .60).

*Impostor syndrome*. We selected 5 of the 20 items from the Clance Impostor Phenomenon Scale [57] and added a face-valid, author-generated item (“Sometimes I feel like a fraud”). Participants responded from 1 (*not at all like me*) to 5 (*very much like me*) (α = .79). Impostor syndrome is common and does not preclude achievement, but it does predict worse psychological well-being, including higher burnout and anxiety [58].

*Academic career preference*. Participants indicated the strength of their preference for an academic job after graduating on a sliding scale, anchored at -5 (*strongly prefer non-academic*), 0 (*equal preference*), and 5 (*strongly prefer academic*).

#### Interpersonal relations

We assessed characteristics related to students’ interpersonal lives, given that social support and integration predict academic persistence at the undergraduate level and in doctoral programs [59, 60].

*Perceived social support*. We measured perceived social support using a single item [61]. Participants indicated how true it was that “There are people I can count on to support me” from 1 (*not at all true*) to 5 (*extremely true*).

*Similarity to colleagues*. A single author-generated item captured perceived similarity to colleagues, “How similar or different to other people in your department do you see yourself?” Responses ranged from 1 (*Very different*) to 6 (*Very similar*).

#### Mental health

Our measure of mental health was adapted from Kessler and colleagues’ scale of psychological distress [62], which assessed how often participants felt nervous, hopeless, restless or fidgety, so depressed that nothing could cheer them up, that everything was an effort, and worthless during the past 30 days, from 0 (*none of the time*) to 4 (*all of the time*). We summed items (α = .85) to create a scale score. Higher scores on the Kessler scale predict lower academic achievement [63].

### Social identity threat

We include measures of stereotype threat and identity interference to assess SIT.

*Stereotype threat*. Adapted from Cohen and Garcia [44], this scale has six items that assess stereotype threat, which we modified for a graduate school context (e.g., “I worry that people in my graduate program will judge me based on what they think of my racial group [people of my gender]”). Students responded from 1 (*strongly disagree*) to 7 (*strongly agree*). Racial (α = .91) and gender (α = .91) stereotype threat were separated. Greater stereotype threat predicts worse academic performance for women and URM students [17].

*Identity interference*. We adapted four items from Settles’ [64] 17-item scale of identity interference, which is when one identity conflicts with another, specifically gender identity and science/researcher identity in this context (e.g., “I feel that other researchers do not take me seriously because of my gender”). Students responded from 1 (*strongly disagree*) to 7 (*strongly agree*). Higher levels of identity interference on this scale have predicted lower self-esteem and lower perceived science performance [64, 65]. We removed one item (“I feel that because of my gender, it is easier for me to fit the definition of a researcher”) to increase consistency from α = .68 to α = .82 (see S6 Appendix).

## Results

### Analytic strategy

**Fig 1:**
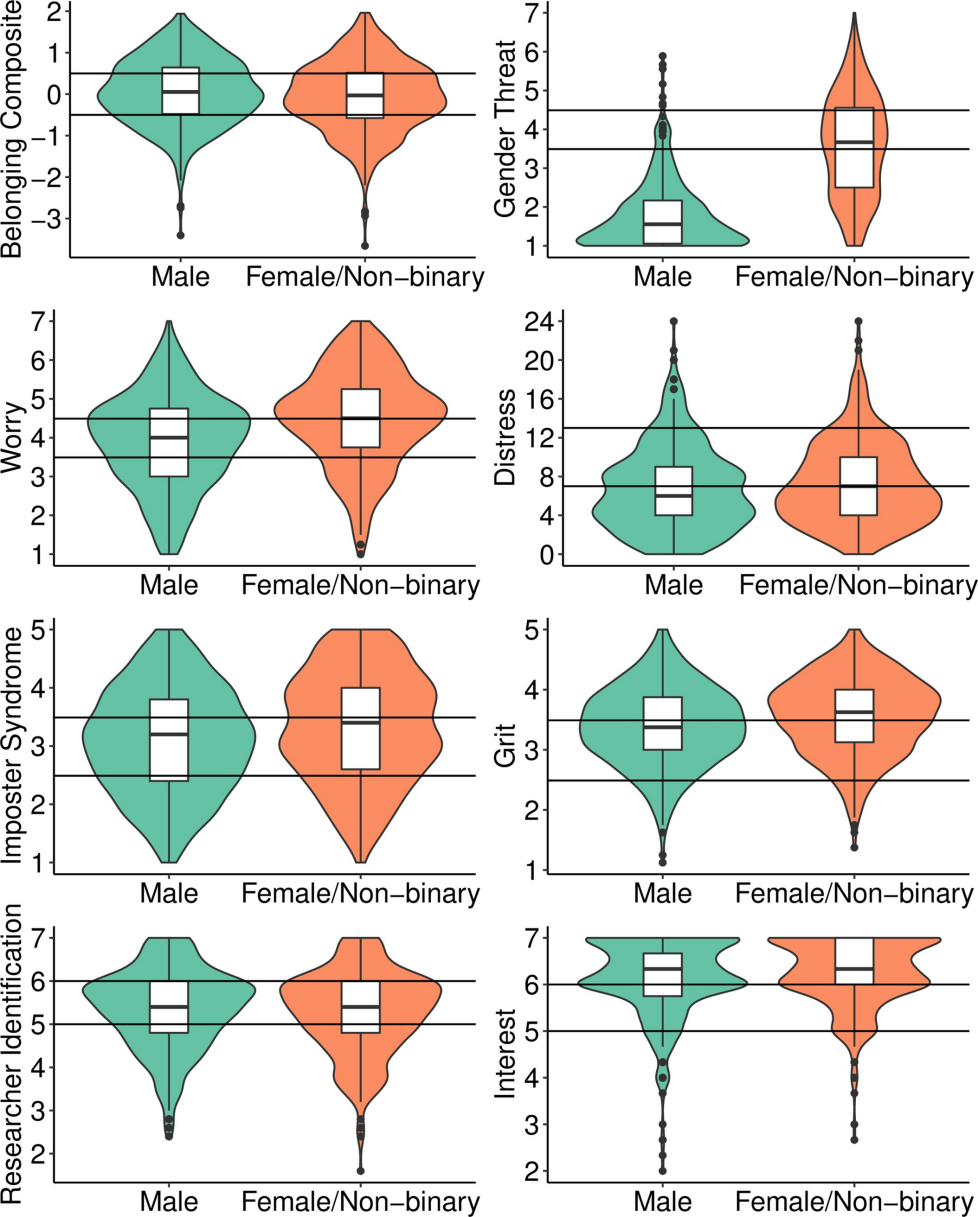
Distribution of indicator items by gender. Horizontal lines indicate cut-offs used to categorize these continuous variables intro trichotomous variables.

For the delineation of classes, we considered several indicator variables and used an iterative process to select a useful set for developing an interpretable and parsimonious model. By design, our goal was to use theory to guide the selection of indicator variables (i.e., those related to psychological threat) and then to empirically derive the best set based on quantitative fit and interpretability of classes. We used LCA instead of latent profile analysis (LPA), a similar technique appropriate when indicators are continuous, because LPA has strict assumptions (e.g., that indicators be normally distributed; see [28]) that create model fit and stability issues in the commonly encountered situation where assumptions are violated. Indeed, we encountered these exact issues, suggesting LPA was not suitable for our data. Thus, we proceeded with LCA, and to do so, we trichotomized the eight indicators that were ultimately selected (see S7 Appendix). Where possible, we created categories based on meaningful scale response options. For instance, for distress we used the cutoff for clinical concern (13) in our trichotomization. For variables that were positively skewed (i.e., interest, researcher identification; see Fig 1), we trichotomized so that each group had a sufficient sample size.

We conducted LCAs with different numbers and combinations of indicator variables related to psychological threat and indicated by past literature as relevant to academic outcomes (see S8 Appendix for considered indicators). We sought indicators that distinguished between classes (i.e., threat profiles) and for a model that was stable and theoretically interpretable. For instance, we eliminated growth mindset and race-based stereotype threat as potential indicators because although theoretically meaningful and relevant, they did not distinguish between classes well. Ultimately, our final model had eight indicators and four classes. Fig 1 displays indicator distributions by gender, given historical gaps in Ph.D. attainment between men and women.

Two of our eight indicator variables, need fulfillment and gender threat, were composite variables suggested by high intercorrelations among predictors that led us to conduct a principal components analysis to try and reduce the number of individual indicator variables (see S9 Appendix for details). Need fulfillment was comprised of academic belonging, graduate school self-efficacy, and psychological need satisfaction. Gender threat was comprised of gender-based stereotype threat and identity interference. Composite variables reduce the number of indicators, which helps facilitate model fit and avoid redundant indicator variables.

To better understand the best fitting LCA model, our analysis strategy next turned to identifying who is in each class, that is, how the classes differed by demographic variables often used as proxies for psychologically threatened groups (e.g., gender). Our goal was to test whether our interpretations of the risk level of the classes mapped onto these demographic variables in expected ways. We chose the following demographic variables to align with this goal: gender, sexual orientation, race/ethnicity, SES, first-generation student status, and international student status. We expected women, sexual minorities, and low-SES/first-generation students to be overrepresented in higher threat, particularly higher SIT, classes (and underrepresented in lower threat classes) given the potential of these groups to face psychological threats beyond what dominant groups encounter. We included race/ethnicity with a similar rationale, but the relatively small and racially heterogeneous URM sample inhibits interpretability. We included international student status for exploratory purposes.

To analyze class differences in demographic variables, we used the BCH procedure within the LCA framework, which is recommended for examining how LCA-derived classes predict outcomes [66]. The BCH procedure uses linear and logistic regression to predict outcomes from class membership accounting for measurement-error weighting associated with assigning people to their most likely class. The procedure can be used cross-sectionally and does not assume an antecedent-consequent structure. The most common alternative approach, classify-analyze, entails assigning individuals to classes without measurement-error weighting, which is contraindicated [67].

Because the BCH technique requires categorical correlates of the classes to be binary, we recoded and converted demographic variables where necessary. We collapsed gender into male (0) and not male (1), given that only 11 students identified exclusively as genderqueer (see Table 1) and the potential for both female and genderqueer identities to suffer heightened psychological threat [68]. Results did not meaningfully differ when only male and female identified students were included. We collapsed the categories for sexual orientation into heterosexual (0) and queer (1), used here to denote all non-heterosexual identities. We compared continuing generation (0) to first-generation students (1). We dichotomized SES, such that students who saw themselves as average or above average, that is, at or above the scale midpoint (≥ 5; 0), were compared to those who saw themselves as below average (< 5; 1). We coded race/ethnicity such that non-URM students (White, Asian, or White/Asian and international; 0) were compared to URM students (at least partially Native American, Hispanic, or Black, and domestic; 1). We coded this way because Native American, Hispanic, and Black students are underrepresented among people with STEM doctoral degrees whereas White and Asian students are not [1]. We did not group international students with URM regardless of race/ethnicity given the unique racial context of the United States, which may not apply to international students. Most international students were Asian, which would preclude them from categorization as URM students regardless. Of course, international students may experience graduate school differently than domestic students, which we tested with a variable comparing domestic students (0) to international students (1).

In addition to examining demographics, we examined campus differences by class to investigate the potential for the local context to influence the proportions of students falling into each class. For instance, perhaps students in a rural setting (e.g., Penn State) worry about finding community and are overrepresented in higher threat classes. To investigate campus, we ran three analyses using the BCH procedure, each analysis with a different binary-coded campus variable (e.g., Penn State and not Penn State, with the latter as the reference group).

The final step in our analysis strategy was to characterize the classes more fully by testing their association with theory-relevant constructs. This step is important because not every construct relevant to academic outcomes can be used as an indicator. For instance, although we did not use race-based stereotype threat as an indicator given it contributed relatively less to a clear class makeup than other variables, the classes may still vary in race-based stereotype threat, which can have implications for overall risk level. Using the recommended BCH technique, we tested for class differences in the continuous indicator variables (i.e., pre-trichotomization), including the components of the two composites, as well as other relevant constructs.

### Model selection

We conducted latent class analyses in Mplus version 8.4 [69] after preparing data files in RStudio version 1.3.1093 [70] using the MplusAutomation package [71]. We evaluated model identification using 1,000 sets of random initial stage starting values and 500 final stage starts. We specified models that varied in the number of indicators, and within those model sets we varied the number of classes and then empirically evaluated relative fit using (1) the Bayesian information criterion (BIC; [72]), (2) the sample-size adjusted BIC (aBIC; [73]), (3) the Akaike information criterion (AIC; [74]), (4) the bootstrapped likelihood ratio test (BLRT; see [75]), and (5) the Vuong-Lo-Mendell-Rubin adjusted likelihood ratio test (VLMR-LRT; see [75]). Lower values for BIC, aBIC, and AIC indicate relatively better balance between parsimony and model fit. We emphasized the BIC and BLRT in particular given evidence showing their unique strength in identifying the ideal number of classes [75]. We also considered absolute model fit (e.g., the *G*^*2*^ likelihood-ratio chi-square statistic; [76]). In addition to evaluating empirical strength, we emphasized theoretical interpretability in model selection [76].

The iterative process of model specification and evaluation ultimately resulted in one interpretable, robust model with eight indicators and four classes. Table 3 presents fit indices for models ranging from 1 to 8 classes using the eight indicators that ultimately proved most important to model specification. Table 3 also presents entropy values for these models. Entropy is a measure of class separation for which higher values indicate greater separability and higher classification utility. Entropy was not used as a model selection statistic, as its utility is in identifying problems with overextraction rather than distinguishing well between the appropriateness of models with different numbers of classes [77]. Table 4 presents average posterior probabilities from the selected 4-class model, which are the average of each individual’s probability of membership in each class. Higher average posterior probabilities reflect greater certainty that members of a class are assigned correctly.

**Table 3:** LCA fit indices.

K	LL	BIC	aBIC	AIC	BLRT *p*	VLMR-LRT *p*	Entropy
1	-8354.490	16820.750	16769.931	16740.980	--	--	--
2	-7872.915	15976.355	15871.540	15811.829	< .0001	< .0001	.714
3	-7731.617	15812.515	15653.705	15563.233	< .0001	.0066	.716
4	-7638.886	**15745.810**	15533.004	15411.772	< .0001	**.0001**	.702
5	-7592.008	15770.810	**15504.009**	15352.016	< .0001	.5328	.700
6	-7564.315	15834.179	15513.382	15330.629	< .0001	.0203	.691
7	-7538.127	15900.560	15525.767	15312.254	**.0128**	.2993	.703
8	-7519.591	15982.244	15553.456	**15309.182**	1.0000	.7636	.724

K = number of classes; LL = log-likelihood; BIC = Bayesian Information Criterion; aBIC = Sample-size adjusted BIC; AIC = Akaike Information Criterion; BLRT = bootstrapped likelihood ratio test; VLMR-LRT = Vuong-Lo-Mendell-Rubin adjusted likelihood ratio test; *p* = *p-*value; Bolded values indicate “best” fit for each respective statistic. Entropy is included in the table for brevity but should not be used as a model selection statistic [77].

**Table 4:** Classification probabilities for the most likely latent class membership (column) by latent class (row).

Class	1	2	3	4
1	**.861**	.104	.035	.000
2	.063	**.797**	.095	.046
3	.020	.086	**.834**	.060
4	.000	.062	.085	**.853**

Bolded values indicate average posterior probabilities.

**Fig 2:**
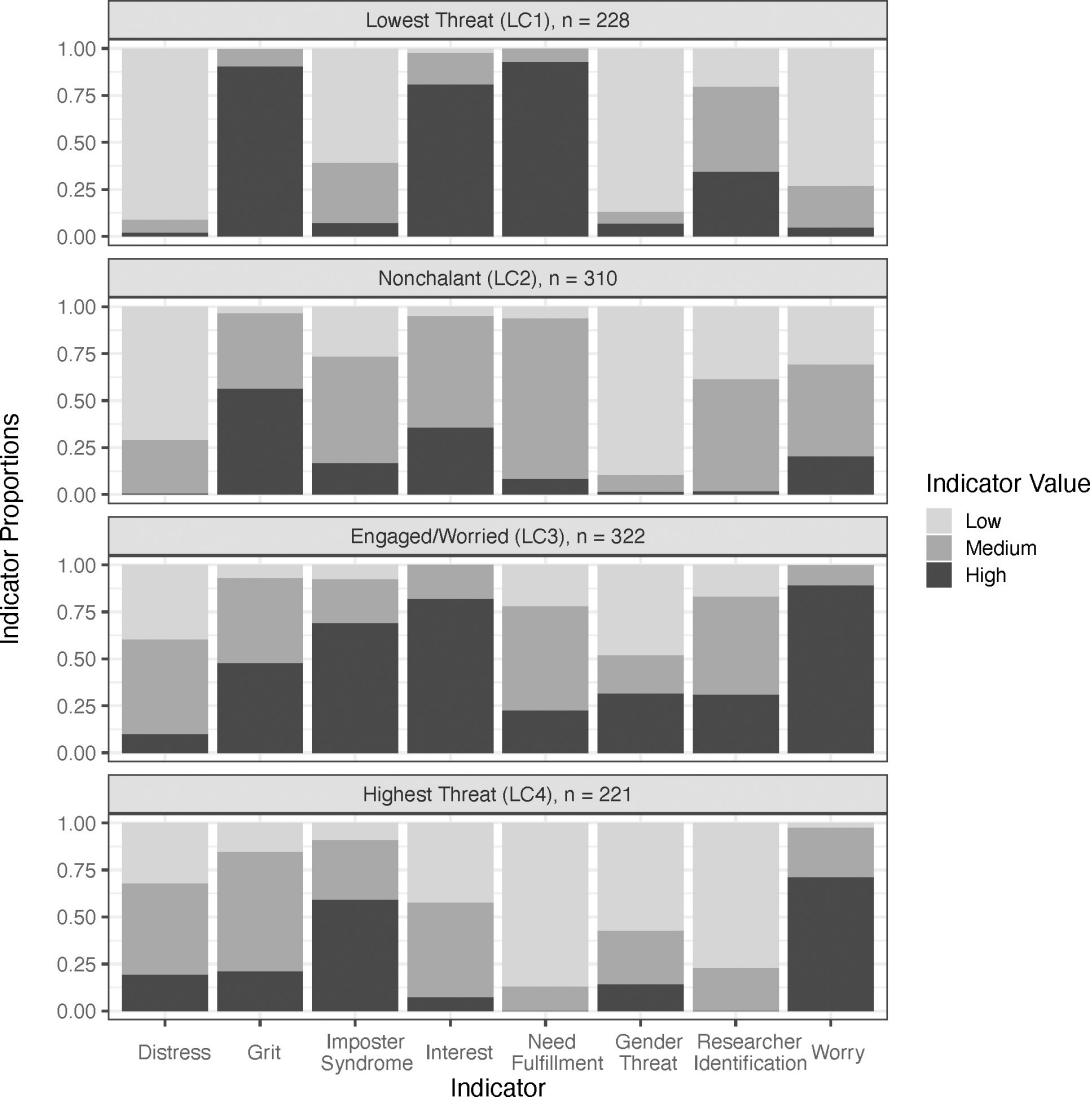
Distribution of indicators by class. LC = Latent Class. “Worry” refers to Academic and Social Concerns.

The two kinds of parameters estimated in LCAs are latent class prevalences (i.e., the proportion of the sample in each class) and item response probabilities, which represent the probability of answering a certain way given membership in each class—these parameters thus reveal the size and core traits of each class (see Fig 2).

### Model description

In this section, we present the description of each of the four classes in the selected model in order from lowest to highest threat based on our interpretations of the classes.

#### *Lowest Threat* class

Class 1, about 21% of the sample, has the least psychological threat. Most Class 1 students are high in need fulfillment, which reflects academic belonging, graduate school self-efficacy, and psychological need satisfaction. The vast majority also report low levels of gender threat, psychological distress, and academic and social concerns, and high levels of grit and interest. Compared to the other classes, Class 1 students report the least impostor syndrome, with over half (59%) having low impostor syndrome. Only 21% reported, on average, a response of less than “somewhat agree” to items assessing researcher identification. Hence, most of these students feel at least moderately identified with their work, and in relative terms, they feel more strongly identified than students in Classes 2 or 4. Overall, Class 1 students appear psychologically prepared for and engaged with graduate school with few concerns. We label Class 1 as *Lowest Threat*.

#### *Nonchalant* class

Class 2, about 29% of the sample, is distinguished by the vast majority of its students scoring (1) in the middle on need fulfillment, (2) low on gender threat, and (3) low on distress. These students face minimal SIT and distress; however, their levels of academic and social concerns vary, with nearly half (45%) not feeling strongly either way about whether they are worried about others perceiving them negatively. Similarly, over half (54%) have moderate levels of impostor syndrome. Class 2 students either report medium or high grit and interest and medium or low researcher identification. Class 2 students vary, but overall do not seem to be particularly high threat based on their response patterns; however, they are not as confident as the *Lowest Threat* class given their responses tend more towards the middle. Given this pattern and the general lack of strong positive or negative feelings, we label Class 2 as *Nonchalant*.

#### *Engaged/Worried* class

Class 3, the largest class at 30% of the sample, is distinguished by having most of its members score high on (1) academic and social concerns and (2) impostor syndrome, but (3) also interest. There is more variation on the need fulfillment composite than in the other classes, although most (55%) members fall in the middle. Although about half of the students in Class 3 report low gender threat, there is more gender threat in this class than in the others, with 30% reporting relatively high levels. Class 3 students tended to be low or medium on distress and medium or high on grit. They are most like the *Lowest Threat* class on researcher identification. Overall, students in Class 3 appear to face some psychological threat, including the highest levels of SIT, but also seem highly engaged in their studies. We label Class 3 as *Engaged/Worried*.

#### *Highest Threat* class

Class 4, the smallest class at about 20% of the sample, consists of students who, for the most part, are low on need fulfillment, high in academic and social concerns, and low in researcher identification. Although the majority (57%) report minimal gender threat, more students in this class than those in the *Nonchalant* or *Lowest Threat* classes experience moderate to high levels of gender threat. Class 4 students vary in their psychological distress, with almost half reporting a moderate amount and 19% reporting clinically concerning levels, which is more than any of the other classes. The majority report high levels of impostor syndrome, with most others reporting medium levels. A higher proportion of Class 4 students than in the other classes scores low on grit, and fewer score high on grit, although most in Class 4 report a medium level. The vast majority of students in Class 4 score low or medium on interest, which is also lower than the other classes. Relatively speaking, students in this class have the most psychological threat. We label Class 4 as *Highest Threat*.

#### Who is in each class?

Table 5 presents class differences on demographics. The BCH procedure provides a chi-square test of the difference between classes on each variable, along with estimated means by class. Because of how the demographic variables are coded, these estimated means represent the proportion of students in a particular demographic category. The BCH procedure also provides significance tests for pairwise comparisons between classes (e.g., does the *Engaged/Worried* class have a significantly higher proportion of women than the *Lowest Threat* class?).

**Table 5:** Proportions of demographic groups by latent class.

Group	Lowest Threat	Nonchalant	Engaged/ Worried	Highest Threat	Base Rate *n* (proportion)
Gender: Female/Genderqueer*	.41 ^d^	.36 ^a, b^	.69 ^a, c, d^	.49 ^b, c^	535 (.49)
Race/ethnicity: Underrepresented Minority	.06	.07	.10	.05	80 (.07)
Sexual orientation: Queer*	.14 ^c^	.12 ^a^	.34 ^a, b, c^	.15 ^b^	212 (.20)
First-generation student: Yes	.23	.15	.23	.20	218 (.20)
Socioeconomic status: Low*	.13 ^a, b, c^	.23 ^a^	.22 ^b^	.26 ^c^	226 (.21)
International student: Yes*	.35 ^c, d^	.55 ^b, d^	.31 ^a, b^	.48 ^a, c^	457 (.42)
Campus: Penn State	.50	.56	.50	.47	553 (.51)
Campus: Columbia	.20	.20	.24	.21	231 (.21)
Campus: Stanford	.30	.23	.26	.33	297 (.27)

Matching letters denote significant pairwise differences at *p* < .05. Asterisks beside variable name indicate overall Chi-square test was significant at *p* < .05. The “Base Rate” column refers to the number/proportion of the entire sample (i.e., across classes) that belongs to the indicated group (e.g., female/genderqueer).

The classes differed by gender, sexual orientation, SES, and international status, but not by first-generation status, race/ethnicity, or campus (see Table 5). We expected relative overrepresentation of potential at-risk social categories (e.g., women) in the higher risk classes, particularly any associated with SIT, and relative underrepresentation in lower risk classes. We did not find this pattern reflected in the *Highest Threat* class, suggesting the need to look beyond group membership when assessing at-risk students, but we did find this in the *Engaged/Worried* class (i.e., the class highest in SIT). The *Engaged/Worried* class has overrepresentation of female/genderqueer and queer identities, suggesting that this class is characterized by relatively high SIT, which coincides with the initial description (i.e., they had relatively high gender threat). The *Lowest Threat* class is underrepresented in terms of low-SES students and somewhat underrepresented in terms of international students; we expected this class to have fewer students who are in at-risk social categories, so the SES finding is unsurprising. The *Nonchalant* class, and to a lesser extent the *Highest Threat* class, have slight overrepresentation of international students. We did not have expectations for international students, but their overrepresentation in the *Highest Threat* class may indicate that some international students are at heightened risk. Overall, results provide some evidence for our predictions on demographic group representation.

### Descriptive information about risk by class

To further understand the implications of class membership for student risk levels, we examined how class membership was related to academic and attrition-relevant proximal characteristics. For these analyses, we used the same approach as we did for the demographic variables, the automated BCH procedure that produces Chi-square results. Class membership was associated with all examined characteristics. Table 6 shows an overview of expected risk level by class and Table 7 shows results. We present the means by class visually in the S10 Appendix.

**Table 6:** A summary of how class membership is associated with descriptive characteristics.

Characteristic Category	Lowest Threat	Nonchalant	Engaged/ Worried	Highest Threat
Academic Preparation & Context	**+**	**+**	**+**	**-**
Academic Identity & Graduate School Attitudes	**+**	0	0	**-**
Interpersonal Relations	**+**	0	0	**-**
Self-Evaluations & Perceived Fit	**+**	0	**-**	**-**
Mental Health	**+**	**+**	**-**	**-**
Social Identity Threat	**+**	**+**	**-**	**-**

A **+** symbol denotes low risk,—denotes high risk, and 0 denotes medium/mixed risk. Risk levels are relative; they were determined by examining statistically significant differences between the classes.

**Table 7:** How class membership is associated with descriptive characteristics.

Variable	Lowest Threat Mean (*SE*)	Nonchalant Mean (*SE*)	Engaged/Worried Mean (*SE*)	Highest Threat Mean (*SE*)
**Academic Preparation & Context**				
Proportion of women in field	0.391 (0.013) ^c, d^	0.344 (0.012) ^b, d^	0.412 (.012) ^a, b^	0.355 (.013) ^a, c^
Years undergrad research	1.995 (0.083) ^c, d^	1.727 (0.083) ^b, d^	2.000 (0.075) ^a, b^	1.760 (0.086) ^a, c^
Years postgrad research	1.347 (0.179) ^b^	1.034 (0.109)	1.146 (0.098) ^a^	0.762 (0.093) ^a, b^
Undergrad research prep	3.609 (0.085) ^c, e, f^	2.949 (0.074) ^b, d, f^	3.343 (0.075) ^a, b, c^	2.697 (0.089) ^a, d, e^
Postgrad research prep	4.174 (0.097) ^b, d, e^	3.817 (0.097) ^c, e^	3.782 (0.094) ^a, b^	2.990 (0.125) ^a, c, d^
English proficiency	4.765 (0.110) ^d, e^	4.404 (0.099) ^b, c, e^	4.831 (0.142) ^a, b^	4.062 (0.123) ^a, c, d^
Has master’s (proportion yes)	0.303 (0.035)	0.376 (0.036) ^a. b^	0.228 (0.030) ^a^	0.257 (0.035) ^b^
**Self-Evaluations & Perceived Fit**				
Neuroticism	2.846 (0.088) ^b, d, e^	3.585 (0.091) ^a, c, e^	4.962 (0.078) ^a, b^	4.860 (0.086) ^c, d^
Self-esteem	4.079 (0.069) ^c, e, f^	3.470 (0.073) ^b, d, f^	2.791 (0.078) ^a, b, c^	2.306 (0.094) ^a, d, e^
Self-efficacy	3.697 (0.029) ^b, d, e^	3.027 (0.035) ^c, e^	3.118 (0.039) ^a, b^	2.435 (0.048) ^a, c, d^
Academic and social concerns	2.680 (0.072) ^c, e, f^	3.590 (0.066) ^b, d, f^	5.353 (0.058) ^a, b, c^	5.007 (0.072) ^a, d, e^
Grit	4.097 (0.038) ^b, d, e^	3.492 (0.044) ^c, e^	3.363 (0.041) ^a. b^	2.961 (0.046) ^a, c, d^
Psychological need satisfaction	5.838 (0.038) ^c, e, f^	5.027 (0.040) ^b, d, f^	4.839 (0.042) ^a, b, c^	4.017 (0.051) ^a, d, e^
Need fulfillment composite	1.089 (0.034) ^b, d, e^	0.025 (0.031) ^c, e^	-0.017 (0.045) ^a, b^	-1.179 (0.050) ^a, c, d^
Academic belonging	6.002 (0.040) ^b, d, e^	5.176 (0.039) ^c, e^	5.152 (0.047) ^a, b^	4.135 (0.054) ^a, c, d^
Belonging uncertainty	3.062 (0.102) ^b, d, e^	4.258 (0.091) ^a, c, e^	5.175 (0.096) ^a, b^	5.047 (0.081) ^c, d^
**Academic Identity & Graduate School Attitudes**				
Interest composite	6.652 (0.037) ^d, e^	6.020 (0.042) ^b, c, e^	6.731 (0.033) ^a, b^	5.078 (0.070) ^a, c, d^
Researcher identification	5.770 (0.071) ^d, e^	5.051 (0.062) ^b, c, e^	5.858 (0.057) ^a, b^	4.387 (0.064) ^a, c, d^
Decision confidence	3.652 (0.038) ^b, d, e^	3.112 (0.043) ^c, e^	3.128 (0.041) ^a, b^	2.622 (0.042) ^a, c, d^
Strength of motivation	5.302 (0.075) ^c, d^	4.540 (0.072) ^b, d^	5.239 (0.067) ^a, b^	4.363 (0.074) ^a, c^
Academic self-control	3.698 (0.063) ^b, d, e^	3.232 (0.062) ^c, e^	3.108 (0.062) ^a, b^	2.620 (0.070) ^a, c, d^
Impostor syndrome	2.295 (0.055) ^b, d, e^	2.822 (0.054) ^a, c, e^	3.885 (0.056) ^a, b^	3.707 (0.065) ^c, d^
Academic career preference	1.992 (0.229) ^c, d^	0.934 (0.211) ^b, d^	1.978 (0.191) ^a, b^	0.857 (0.228) ^a, c^
**Interpersonal Relations & Perceived Fit**				
Perceived social support	4.378 (0.063) ^d, e^	4.001 (0.063) ^b, c, e^	4.223 (0.059) ^a, b^	3.434 (0.076) ^a, c, d^
Similarity to colleagues	4.183 (0.102) ^c, e^	4.024 (0.091) ^b, d^	3.632 (0.096) ^a, b, c^	3.184 (0.099) ^a, d, e^
**Mental Health**				
Distress	3.768 (0.239) ^c, e, f^	5.254 (0.238) ^b, d, f^	8.822 (0.269) ^a, b, c^	10.434 (0.364) ^a, d, e^
**Social Identity Threat**				
Stereotype threat- race	1.904 (0.087) ^c, e, f^	2.466 (0.095) ^b, d, f^	2.902 (0.109) ^a, b, c^	3.341 (0.111) ^a, d, e^
Stereotype threat- gender	1.965 (0.084) ^b, d^	2.133 (0.079) ^a, c^	3.427 (0.103) ^a, b^	3.186 (0.105) ^c, d^
Gender threat composite	2.017 (0.091) ^c, e^	1.968 (0.074) ^b, d^	3.610 (0.112) ^a, b, c^	3.026 (0.107) ^a, d, e^
Identity interference	2.203 (0.098) ^c, e^	2.129 (0.089) ^b, d^	3.551 (0.112) ^a, b, c^	2.962 (0.113) ^a, d, e^

Matching letters denote significant pairwise differences at *p* < .05. Overall Chi-square tests for all variables were significant at *p* < .05.

### *Lowest Threat* class

Overall, at matriculation, *Lowest Threat* students fared better than the other classes.

*Lowest Threat* students were very academically prepared. For instance, compared to others, they found their prior research experience most helpful in preparing them for graduate school. These students also reported the most positive attitudes towards graduate school and academia; they felt the surest about pursuing a Ph.D. and were highly motivated to finish. They similarly had the most positive perceptions of their interpersonal relations. Aligned with their initial description as self-assured, *Lowest Threat* students also had the most positive self-evaluations and perceptions of fit, with the least neuroticism and uncertainty about belonging, and the highest self-efficacy, self-esteem, and belonging. These students also reported the least distress and race-based stereotype threat and low levels of gender-based SIT.

Taken together, results support the notion that *Lowest Threat* students are at low risk of adverse outcomes like attrition and may foreshadow relatively positive academic outcomes.

#### *Nonchalant* class

Students in the *Nonchalant* class tend to fall in the middle in terms of threat compared to the other classes but are more like the *Lowest Threat* class than the other two classes.

*Nonchalant* students were relatively well-prepared; for instance, this class had the highest rate of students entering with master’s degrees. However, among non-native speakers, English proficiency was lower than *Lowest Threat* and *Engaged/Worried* students, which could cause challenges (e.g., with social integration or writing). *Nonchalant* students had mixed attitudes towards graduate school and academia; they felt less sure about pursuing a Ph.D. than *Lowest Threat* students but more sure than *Highest Threat* students, and they were less motivated to finish than *Lowest Threat* and *Engaged/Worried* students. *Nonchalant* students also had mixed characteristics regarding interpersonal relations. For instance, they perceived less social support than *Lowest Threat* and *Engaged/Worried* students but felt as similar to their colleagues as *Lowest Threat* students. *Nonchalant* students tended to be in between the *Lowest Threat* and *Highest Threat* classes on self-evaluations and perceived fit; on all self-evaluations, *Nonchalant* students fared better than or similarly to *Engaged/Worried* students. These students also reported more distress and stereotype threat than *Lowest Threat* students but less than the other classes.

Given these results, we would expect *Nonchalant* students to be at lower risk of adverse outcomes than *Engaged/Worried* and *Highest Threat* students but not to be entirely risk-free.

#### *Engaged/Worried* class

Overall, results support the assessment of the *Engaged/Worried* class as the class facing the second-highest levels of psychological threat.

*Engaged/Worried* students were fairly well-prepared academically. Students in this class had a similar amount of undergraduate research experience as *Lowest Threat* students but felt significantly less prepared by it. This discrepancy may indicate that *Engaged/Worried* students feel less confident than their objective qualifications warrant. This finding may be a sign of SIT, particularly considering that the majority of this class is female, and is consistent with women (vs men) tending to be underconfident in STEM fields [39]. Indeed, *Engaged/Worried* students reported the most gender-based SIT, although the average was below the scale midpoint. *Engaged/Worried* students also had mixed attitudes towards graduate school and academia and mixed perceptions of interpersonal relations that further suggest their high engagement with graduate school alongside self-doubts. For instance, they reported higher motivation to finish than *Nonchalant* and *Highest Threat* students and perceived similarly high levels of social support as *Lowest Threat* students but had the most impostor syndrome (above the scale midpoint) and saw themselves as less like their colleagues than *Lowest Threat* and *Nonchalant* students (albeit above the midpoint). *Engaged/Worried* students also had somewhat poor self-evaluations, perceptions of fit, and mental health; they reported the most academic and social concerns (above the scale midpoint) and fared worse than *Nonchalant* and *Lowest Threat* students on self-esteem (below the midpoint) and distress.

Overall, *Engaged/Worried* students seem to be struggling more with threat than *Nonchalant* students, although it is possible that their high engagement is protective against other sources of threat, or that their high neuroticism and other forms of worry are beneficial (e.g., they motivate rather than immobilize). However, the relatively high threat levels of these students at the beginning of graduate school may predict suboptimal academic or well-being outcomes.

#### *Highest Threat* class

Results suggest that *Highest Threat* students are struggling at the start of graduate school.

*Highest Threat* students were pursuing their Ph.D.’s in fields that are on average ~36% female, despite the class being evenly split by gender (51% male). Thus, female students in this class may tend to be underrepresented in their fields of study, which can create SIT. Indeed, *Highest Threat* students reported relatively high levels of gender-based SIT; they also reported the highest levels of race-based stereotype threat (albeit below the scale midpoint). Additionally, non-native English speakers in this class reported the least English proficiency, which, as noted previously, could pose challenges. *Highest Threat* students were also least prepared, with the least postgraduate prior research experience and the lowest sense of preparedness from prior experience. They also reported the most negative attitudes towards graduate school/academia and the most negative interpersonal relations. For instance, they felt the least interest in their work, the most doubt about pursuing a Ph.D., the least supported, and the least like their colleagues. These students also evaluated themselves the most negatively and as having the worst fit, with the lowest self-esteem, self-efficacy, and sense of belonging. Moreover, although their average did not reach clinical thresholds, *Highest Threat* students reported more distress than others. Notably, although some class differences are relative, others may be concerning on a more absolute level: *Highest Threat* students scored above the scale midpoints on impostor syndrome, neuroticism, academic and social concerns, and belonging uncertainty, and they scored below the midpoints on feelings of preparedness, academic self-control, similarity to colleagues, self-efficacy, and self-esteem.

## Discussion

Using latent class analysis (LCA), we identified four classes of incoming Ph.D. students that varied in psychological threat experiences. Even among this elite group of students—who represent a critical population for the advancement of knowledge—there is a meaningful proportion who begin graduate school in a seemingly tenuous psychological state. We highlight the possibility of identifying such students early and tailoring interventions to prevent attrition.

We found that two classes reported fairly positive psychological states at matriculation. Students in the *Lowest Threat* class consistently reported the most auspicious characteristics and given their all-around low-threat psychological state, seem to be at low risk of adverse outcomes. Students in the *Nonchalant* class were like those in the *Lowest Threat* class but had less propitious characteristics. *Nonchalant* students were neither greatly worried about nor greatly engaged in graduate school, although they had generally more positive than negative characteristics. For instance, they were well-prepared for graduate school and had positive self-evaluations but had mixed attitudes towards graduate school. They also reported having relatively low social support, perhaps linked to the overrepresentation of international students in this class. International students may face more difficulties integrating socially than non-international students, given cultural differences, geographic distance from family, and for some, language barriers [78]. Overall, we expect *Nonchalant* students to be at low risk of attrition from psychological threat, but it will be important to follow their trajectory to identify which forms of psychological threat are more or less impactful for Ph.D. student persistence.

The other two classes tended to report more negative psychological states. The *Engaged/Worried* class was characterized by reports of high interest in and engagement with graduate school but also high threat, with concerns about fitting in and being judged. Although this class demonstrated high preparation, their relatively high negative affect, uncertainty, SIT, and distress may portend heightened attrition risk. In general, these students evaluated themselves rather negatively, which likely does not reflect their competencies given their prior research experience and acceptance into their programs. We expected female, queer, first-generation, and URM students to face more SIT than other students and for them to be overrepresented in higher threat classes; given these expectations and our similar pre-registered hypotheses, we found it unsurprising that the *Engaged/Worried* class had the most women and queer students and reported the most SIT. Although not the highest threat class, the *Engaged/Worried* class appears to be relatively high risk based on students’ tendency to report inauspicious characteristics and psychological attributes. It is unclear whether these students’ high engagement would offset other threats. One possibility is that these students will thrive in supportive environments but struggle in unsupportive environments without intervention. Thus, this class could be an important indicator of risk used to target intervention efforts.

The *Highest Threat* class had the least auspicious scores on most measures. At matriculation, *Highest Threat* students were least prepared, reported negative attitudes and self-evaluations, worried about their relationships and fit, felt highly distressed, and experienced relatively high SIT. They tended to be in fields with fewer women despite the class being split evenly by gender, which may contribute to their relatively high gender-based SIT. They had the most negative characteristics in most domains, and differences were not just relative; their average scores on many threat-relevant variables fell on the unfavorable side of the scale midpoint. We thus expect these students are at the highest risk of attrition. Accordingly, we suggest these students might have the most to gain from intervention and might particularly benefit from one that targets the breadth of their threats. For instance, they may benefit from an approach that targets preparedness *and* belonging, like a summer program that has built confidence and community for incoming undergraduates [79] and from social psychological interventions meant to bolster psychological resources.

Notably, the *Highest Threat* class was not overrepresented in terms of high-risk social categories. This suggests the importance of looking beyond group membership when designing ways to assess threat and reduce attrition for STEM Ph.D. students; indeed, the approach we took with LCA, instead of using social categories as threat proxies, seems to provide much nuance.

Moreover, our findings shed light on how specific psychological theories might provide helpful frameworks for investigating the experiences of doctoral students. For instance, the characterization of the *Engaged/Worried* class highlights that SIT theory is applicable among doctoral students and underscores the importance of investigating disparities by demographic group. At the same time, however, our results regarding the *Highest Threat* class demonstrate that students might be at-risk even if they do not face SIT specifically. Moreover, we find that our results are interesting to consider in the context of the Zone Model of Threat theory [30], which draws from the expectancy-value theory of achievement motivation [7] and suggests two key dimensions by which students can be identified for the purpose of tailoring interventions: (1) how much students value a domain and (2) what students expect about their ability to succeed in that domain. Binning and Browman [30] suggest that students who highly value a domain but feel worried about succeeding in it (i.e., one of the four threat zones) would benefit most from interventions that aim to better align student expectancies with motivations. We suggest that the *Engaged/Worried* class aligns with this threat zone, as students in this class were highly engaged with their studies but simultaneously worried about their ability to succeed. As such, our future work with SAGES data examining how the *Engaged/Worried* and other classes respond to expectancy-enhancing social psychological interventions will be an interesting test of the Zone Model of Threat theory.

Overall, our findings highlight the importance of attending to Ph.D. students’ psychological experiences. Ph.D. students are important for universities and the future of research, as they become responsible for scientific discovery, theoretical innovation, and teaching the next generation. They are among the top students in the world, and their aptitude is thoroughly vetted before admission. Thus, it is distressing if these highly qualified scientific leaders leave, not due to inability, but due to psychological barriers. We found that two classes, half of the sample, reported relatively high psychological threat in many domains relevant to academic persistence, with one of these classes faring clearly worse than the rest at the start of their studies. In following up on these students, we will use the baseline threat profiles identified here to examine psychological changes throughout doctoral education based on differing starting points by class, as well as how different types of students respond to two distinct social-psychological interventions. Ultimately, it will be important to examine the utility of these classes for identifying which students may be more likely to attrit in the absence of intervention.

Limitations include generalizability constraints, in part due to the features of LCA, which can be difficult to generalize, as class construction is sample-specific. Although we conducted robustness analyses by cohort and campus that support generalizability (see S11 Appendix), we cannot conclude that the same classes would emerge in another sample or setting. We also had to trichotomize some indicator variables in sample-specific ways (i.e., using observed distributions), which could exacerbate overfitting. Similarly, our sample is specifically comprised of doctoral students at three relatively elite universities, and even if our findings are generalizable to similar universities, it is possible that our findings may not generalize to all universities. However, there is evidence to suggest that more prestigious universities produce disproportionately high numbers of faculty [80] and thus, regardless of how well our findings can speak to all types of university, our research may have important implications for the future of knowledge production and teaching.

Future research would benefit from finding a simpler way of identifying at-risk students that may be more easily implemented, even if it may lose some of the nuance of LCA. One option would be to reverse-engineer the classes by finding a way to reduce the number of indicators while maintaining sufficient complexity to recapitulate relevant outcomes and identify at-risk students. As we examine how these classes fare over time, we can better identify which features of each class are most critical to measure. Despite limitations, our research provides a framework for identifying the types of Ph.D. students who may be psychologically threatened at matriculation.

## Conclusion

Using LCA among a large sample of incoming Ph.D. students, we identified four psychological threat profiles that may portend different risk levels of negative psychological and academic outcomes. Although future work is needed to determine long-term implications, it is notable that a substantial number of Ph.D. students face relatively high psychological threat at matriculation. These results are important for educators, administrators, and policymakers to help determine the support some students may need to ensure their—and in turn society’s—success.

## Supporting information

S1 Appendix Unrestricted 4-class LCA using all available data. (DOCX)

S2 Appendix Comparing sample demographics to population demographics. (DOCX)

S3 Appendix Recruitment details and timing for each campus. (DOCX)

S4 Appendix International student status determination. (DOCX)

S5 Appendix Field classifications for the percentage of women by field. (DOCX)

S6 Appendix Descriptive information and results for individual items. (DOCX)

S7 Appendix Trichotimization details. (DOCX)

S8 Appendix Indicators considered for LCA. (DOCX)

S9 Appendix Composite indicator variables. (DOCX)

S10 Appendix Visual presentation of characteristic means by class. (DOCX)

S11 Appendix LCA subsample (robustness) analyses. (DOCX)
