# Supplementary material for: A latent class analysis approach to the identification of doctoral students at risk of attrition
Source: PLoS One. 2023 Jan 13;18(1):e0280325. doi: 10.1371/journal.pone.0280325 (PMC9838860; doi:10.1371/journal.pone.0280325)
Supplement: S1 Appendix — (DOCX) [file pone.0280325.s001.docx]

**S1 Appendix.** **Unrestricted 4-Class LCA Using All Available Data.**

For our primary analyses, we decided to only use responses from students who reached the end of and submitted the baseline survey, and we thus report those results in the main text. In Figure A, we present the main results of the latent class analysis using all available data—that is, including students who did not finish the baseline survey but who may have responded to some or all of the indicators in the LCA. Adding these 44 participants (total *N* = 1,125) did not change the latent classes that emerged, and the percentage of people in each class was similar.

**Figure A. Item Distribution by Class Including *n* = 44 who did not Finish the Baseline Survey (*N* = 1,125).**


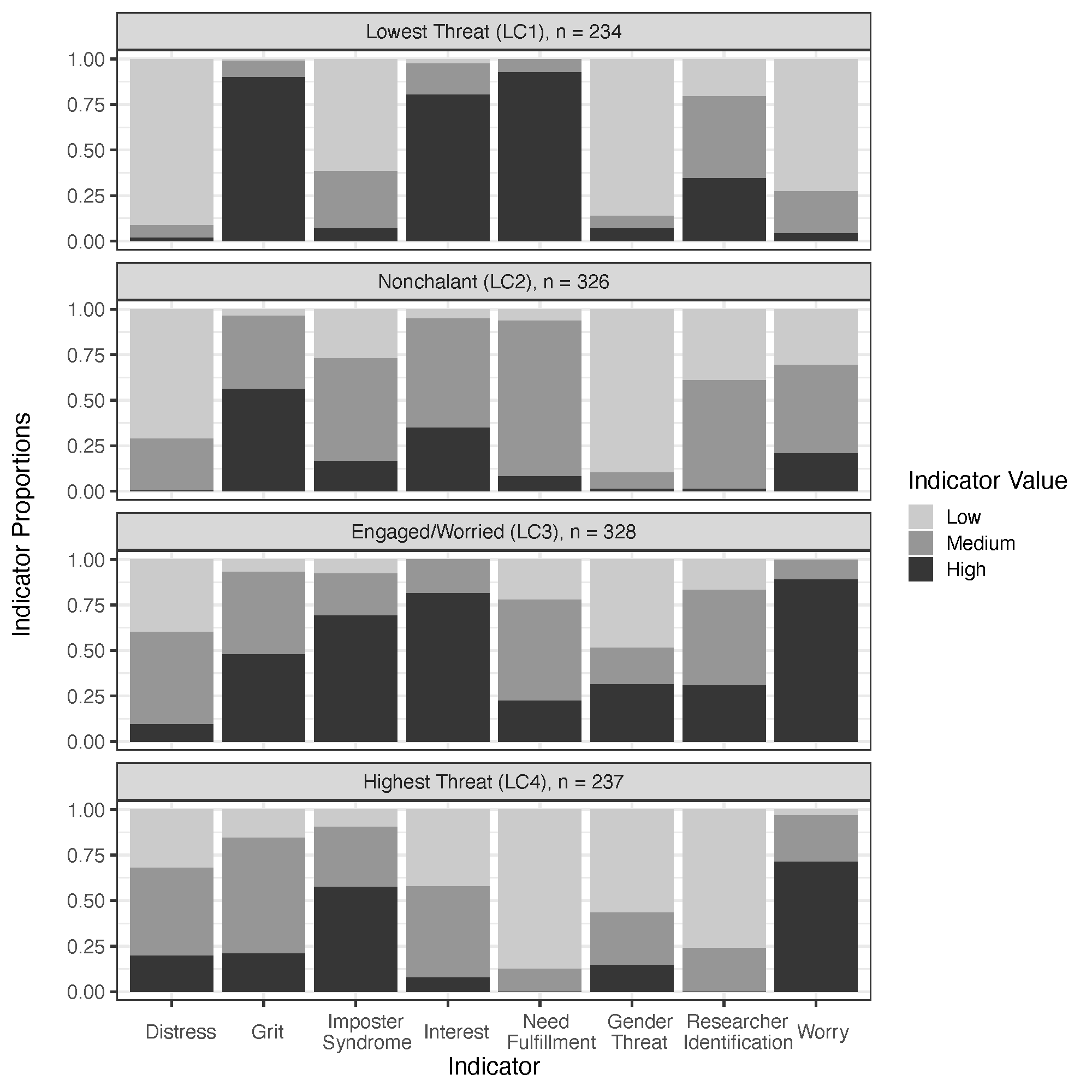
 LC = Latent Class.
