# Supplementary material for: A latent class analysis approach to the identification of doctoral students at risk of attrition
Source: PLoS One. 2023 Jan 13;18(1):e0280325. doi: 10.1371/journal.pone.0280325 (PMC9838860; doi:10.1371/journal.pone.0280325)
Supplement: S2 Appendix — (DOCX) [file pone.0280325.s002.docx]

**S2 Appendix. Comparing Sample Demographics to Population Demographics.**

For Penn State (both cohorts) and Cohort 1 at Stanford, we were able to compare our sample to population data using information that university administrators provided about the gender/sex (male, female) and underrepresented minority (URM) status (domestic URM, domestic non-URM, and international) of the entire incoming classes in our targeted fields. Because both survey responses and the student population data were anonymous, we were unable to determine which incoming students completed the survey and therefore could not directly compare students who participated in this study with students who did not participate. However, we were able to conduct goodness-of-fit tests comparing our sample to the population of the entire incoming class (for Penn State and for Stanford Cohort 1). Demographic data about incoming Ph.D. students were not available at Columbia or for the second cohort at Stanford.

At Penn State, we found that the gender/sex distribution differed between our sample and the population, χ^2^(1) = 10.08, *p* = .002, with a slightly higher percentage of female students in our sample (50.09%) than in the population (43.35%). The URM distribution was also different between the Penn State sample (7.05% URM, 48.46% non-URM, 44.48% international) and population (9.51% URM, 42.71% non-URM, 47.78% international), χ^2^(2) = 9.06, *p* = .011, with slightly lower percentages of URM and international students in our sample.

For Stanford Cohort 1, we conducted analyses with two different subgroups. One subgroup comprised students in targeted engineering fields, who we were able to recruit directly for SAGES, and the other subgroup comprised students in targeted non-engineering STEM fields, who we were not able to recruit directly given logistical issues at Stanford (i.e., we were unable to email incoming students in these fields, although we still asked the relevant departmental administrators to forward our invitation to participate in SAGES to their incoming Ph.D. students). We found similar results in both subgroups, namely that gender distributions significantly differed but URM distributions did not (*p*’s > .05). Within the engineering fields, there was a higher percentage of female students in our sample (55.38%) than in the population (32.29%), χ^2^(1) = 15.85, *p* < .001. Within the non-engineering STEM fields, our sample similarly had a higher percentage of female students (58.23%) than the population (38.33%), χ^2^(1) = 13.23, *p* < .001.

Penn State, but not the other universities, also provided summary data on GRE scores of incoming Ph.D. students in our targeted fields, allowing us to conduct t-tests comparing sample to population means. Quantitative scores were similar between the Penn State sample and entering classes (*p* > .05). However, we did find statistically significant differences for verbal and writing scores. The mean verbal percentile was slightly higher in the Penn State sample (73.37) than in the respective population (68.35), *t*(382) = 4.68, *p* < .001. We investigated this discrepancy further by looking within subgroups, and we found that a discrepancy exists for female, non-URM, and international students, but not for male or URM students (*p*’s > .05). Within female students, the mean verbal percentile is higher for the sample (77.04) than the population (69.69), *t*(182) = 5.63, *p* < .001. Within non-URM students, the discrepancy in mean verbal percentile is smaller between the sample (81.04) and population (78.41) but in the same direction, *t*(196) = 2.19, *p* = .029. Within international students, the discrepancy in mean verbal percentile is similarly slight between the sample (63.27) and population (59.34) but also in the same direction, *t*(154) = 2.21, *p* = .029. A similar overall divergence emerged for GRE writing percentile, with the mean writing percentile being higher in the sample (63.81) than in the population (56.13), *t*(373) = 5.42, *p* < .001; this divergence is statistically significant for all subgroups examined (female, male, URM, non-URM, and international students).

In sum, the comparisons we were able to make between sample and population suggest that our Penn State and Cohort 1 Stanford sample were reasonably representative of the population, although slightly overrepresented in terms of female students, with a larger discrepancy for Stanford than for Penn State. Additionally, our Penn State sample was slightly underrepresented in terms of URM and international students. When we compared GRE scores between the Penn State sample and population, we found that our sample had similar quantitative scores but higher verbal and writing scores, the verbal divergence being driven by female, non-URM, and international students in our sample reporting higher scores. It is possible that students with particularly low verbal and writing GRE scores were less likely to complete the baseline survey, then, although it is also possible that students with particularly low verbal and writing GRE scores (in certain demographic groups) were less likely to report their GRE scores to SAGES or that students self-reported inflated scores. Given missing data in our sample for GRE scores and the limitations of self-report data, it is unclear exactly the cause for discrepancies.
