# Supplementary material for: A latent class analysis approach to the identification of doctoral students at risk of attrition
Source: PLoS One. 2023 Jan 13;18(1):e0280325. doi: 10.1371/journal.pone.0280325 (PMC9838860; doi:10.1371/journal.pone.0280325)
Supplement: S3 Appendix — (DOCX) [file pone.0280325.s003.docx]

**S3 Appendix. Recruitment Details and Timing.**

Here we present recruitment details specifically for each university, given slight variations by campus.

At Penn State, the Graduate School sent a recruitment email written by the research team to incoming doctoral students that had an introduction from the dean. The dean’s message noted the Graduate School’s support for the study but emphasized the independence of the Graduate School and assured students that their participation would not be known by the University or affect their graduate career in any way. The recruitment email was sent to every new doctoral student in 7 STEM-focused colleges (Agricultural Sciences, Earth and Mineral Sciences, Engineering, Information Sciences and Technology, the Eberly College of Science, the Intercollege Graduate Programs, and the College of Medicine). To create an exploratory non-STEM comparison group (although STEM vs non-STEM differences were not of interest in the present work, and we thus included students across fields in our analytic sample), the email was also sent to the relatively smaller incoming classes in the College of Arts and Architecture and the College of the Liberal Arts and the Departments of Biobehavioral Health and Human Development and Family Studies (within the College of Health and Human Development). The recruitment email was sent to students approximately one month before the Fall semester began, with two reminder emails in the following week. Because recruitment emails were all sent through the Graduate School, the research team was not aware of any details of individual non-participating students, and the Graduate School was not aware of whether students had opted to participate. The Graduate School provided the research team limited demographic data about the entire incoming doctoral student cohorts by doctoral field. Members of the research team also attended in-person orientation events for doctoral students where they handed out fliers to remind students about the study in case they missed the emails. Students outside of the targeted fields were allowed to participate if they became aware of the study from recruitment at the orientation events.

At Columbia, with assistance from their respective deans, recruitment emails were sent to all new doctoral students in the Graduate School of Arts and Sciences and the School of Engineering and Applied Science. Emails were not substantively different from those at Penn State but were edited minimally to be appropriate for a different university. Initial emails were sent during the first or second week of the Fall semester (second week for Cohort 1, first for Cohort 2) with two reminders sent the following week.

At Stanford, recruitment emails were sent to all new engineering doctoral students during the second week of the Fall quarter, with two reminders sent the third week. Because at Stanford we did not have direct access to incoming STEM doctoral students outside of engineering, the research team sent the recruitment email to directors of graduate studies and department chairs in STEM departments in the School of Humanities and Sciences with a request to forward the message to incoming doctoral students. We also recruited Stanford students at an in-person orientation event for new graduate students where the research team handed out fliers and provided information about the study.
