# Supplementary material for: A latent class analysis approach to the identification of doctoral students at risk of attrition
Source: PLoS One. 2023 Jan 13;18(1):e0280325. doi: 10.1371/journal.pone.0280325 (PMC9838860; doi:10.1371/journal.pone.0280325)
Supplement: S4 Appendix — (DOCX) [file pone.0280325.s004.docx]

**S4 Appendix. International Student Status Determination.**

To determine students’ international status, we asked students their country of birth, citizenship status, and for U.S. citizens or permanent residents not born in the U.S., the age at which they entered. We also considered whether participants considered the U.S. to be their home country, which we asked students who indicated that they were U.S. citizens or permanent residents in a later survey, not the baseline—thus, we only had the information for a subset of participants who continued participating in the longitudinal aspect of the study. We asked about home country because some people may be U.S. citizens or permanent residents without having lived in the U.S. If participants were born in the U.S. (including Puerto Rico) or if they (1) reported being a U.S. citizen or permanent resident and (2) considered the U.S. their home country, we classified them as domestic; otherwise, we classified them as international. If we did not have data on whether they considered the U.S. their home country, but they were U.S. citizens or permanent residents, we classified them as domestic if they entered the U.S. at ten years old or younger and international otherwise. We classified people who were neither U.S. citizens nor permanent residents as international.
