# Supplementary material for: A latent class analysis approach to the identification of doctoral students at risk of attrition
Source: PLoS One. 2023 Jan 13;18(1):e0280325. doi: 10.1371/journal.pone.0280325 (PMC9838860; doi:10.1371/journal.pone.0280325)
Supplement: S5 Appendix — (DOCX) [file pone.0280325.s005.docx]

**S5 Appendix. Field Classifications for the Percentage of Women by Field.**

For the percentage of women in field variable, we matched fields of study among SAGES participants to a detailed listing of fields of study used in the NSF Survey of Earned Doctorates (SED) [1]. Most matching was straightforward upon examination of the detailed SED fields. When a specific field was not listed in the SED stimuli, we used materials like a doctoral program’s website to inform our classifications. We present the results of this matching process in Table A.

**Table A. How SAGES Fields Were Categorized Using NSF Classifications to Determine Women by Field.**

| **Fields Listed by SAGES Participants** | **Matching to NSF Field Categories** |
| --- | --- |
| Acoustics | Physics and astronomy |
| Aeronautics and Astronautics | Aerospace, aeronautical, and astronautical engineering |
| Aerospace Engineering | Aerospace, aeronautical, and astronautical engineering |
| Agricultural and Biological Engineering | Other engineering |
| Agricultural and Extension Education | Teaching fields |
| Agronomy | Agricultural sciences and natural resources |
| Anatomy | Biological and biomedical sciences |
| Animal Science | Agricultural sciences and natural resources |
| Anthropology | Anthropology |
| Applied Linguistics | Other social sciences |
| Applied Physics | Physics and astronomy |
| Applied Physics and Applied Mathematics | Materials science engineering |
| Architectural Engineering | Other engineering |
| Architecture | Other humanities and arts |
| Art Education | Teaching fields |
| Astronomy | Physics and astronomy |
| Astronomy and Astrophysics | Physics and astronomy |
| Biobehavioral Health | Health sciences |
| Biochemistry, Microbiology, and Molecular Biology | Biological and biomedical sciences |
| Bioengineering | Bioengineering and biomedical engineering |
| Bioinformatics and Genomics | Biological and biomedical sciences |
| Biological Sciences | Biological and biomedical sciences |
| Biology | Biological and biomedical sciences |
| Biomedical Engineering | Bioengineering and biomedical engineering |
| Biomedical Informatics | Biological and biomedical sciences |
| Biomedical Sciences | Biological and biomedical sciences |
| Biophysics | Biological and biomedical sciences |
| Biostatistics | Biological and biomedical sciences |
| Cellular Physiology and Biophysics | Biological and biomedical sciences |
| Cellular, Molecular, and Biomedical Studies | Biological and biomedical sciences |
| Chemical Engineering | Chemical engineering |
| Chemical Physics | Physics and astronomy |
| Chemistry | Chemistry |
| Civil and Environmental Engineering | Civil engineering |
| Civil Engineering | Civil engineering |
| Civil Engineering and Engineering Mechanics | Civil engineering |
| Classics | Letters |
| Communication Arts and Sciences | Other humanities and arts |
| Comparative Literature | Letters |
| Computational and Mathematical Engineering | Other engineering |
| Computer engineering | Electrical, electronics, and communications engineering |
| Computer Science | Computer and information sciences |
| Computer Science and Engineering | computer and information sciences |
| Criminology | Other social sciences |
| Curriculum and Instruction | Education research |
| Developmental and Psychological Sciences | Psychology |
| Earth and Environment Engineering | Other engineering |
| Earth and Environmental Sciences | Geosciences, atmospheric sciences, and ocean sciences |
| Earth System Science | Geosciences, atmospheric sciences, and ocean sciences |
| Ecology | Biological and biomedical sciences |
| Ecology, Evolution, and Environmental Biology | Biological and biomedical sciences |
| Economics | Economics |
| Educational Policy | Education administration |
| Electrical Engineering | Electrical, electronics, and communications engineering |
| Energy and Mineral Engineering | Other engineering |
| Energy, Environmental, and Food Economics | Agricultural sciences and natural resources |
| Engineering Science and Mechanics | Mechanical engineering |
| English | Letters |
| Entomology | Agricultural sciences and natural resources |
| Environmental Engineering | other engineering |
| Food Science | Agricultural sciences and natural resources |
| Forest Resources | Agricultural sciences and natural resources |
| Genetics and Development | Biological and biomedical sciences |
| Geography | Geosciences, atmospheric sciences, and ocean sciences |
| Geophysics | Geosciences, atmospheric sciences, and ocean sciences |
| Geosciences | Geosciences, atmospheric sciences, and ocean sciences |
| German | Foreign languages and literature |
| Higher Education | Education administration |
| Hospitality Management | Business management and administration |
| Human Development and Family Studies | Health sciences |
| Industrial Engineering | Industrial and manufacturing engineering |
| Industrial Engineering and Operations Research | Industrial and manufacturing engineering |
| Informatics | Computer and information sciences |
| Integrative and Biomedical Physiology | Biological and biomedical sciences |
| Learning Sciences and Technology Design | Education research |
| Management Science and Engineering | other engineering |
| Materials Science and Engineering | Materials science engineering |
| Mathematics | Mathematics and statistics |
| Mathematics Education | Teaching fields |
| Mechanical Engineering | Mechanical engineering |
| Meteorology and Atmospheric Science | Geosciences, atmospheric sciences, and ocean sciences |
| Microbiology and Immunology | Biological and biomedical sciences |
| Molecular, Cellular, and Integrative Biosciences | Biological and biomedical sciences |
| Music Education | Teaching fields |
| Neurobiology and Behavior | Biological and biomedical sciences |
| Neuroscience | Biological and biomedical sciences |
| Neuroscience (Life sciences) | Biological and biomedical sciences |
| Neurosciences | Biological and biomedical sciences |
| Nuclear Engineering | Other engineering |
| Nutritional and Metabolic Biology | Biological and biomedical sciences |
| Nutritional Sciences | Health sciences |
| Pathobiology | Biological and biomedical sciences |
| Pathobiology and Molecular Medicine | Biological and biomedical sciences |
| Pharmacology and Molecular Signaling | Biological and biomedical sciences |
| Philosophy | Other humanities and arts |
| Physics | Physics and astronomy |
| Physiology & Cellular Biophysics | Biological and biomedical sciences |
| Plant Biology | Biological and biomedical sciences |
| Plant Pathology | Agricultural sciences and natural resources |
| Political Science | Political science and government |
| Psychology | Psychology |
| Race, Inequality and Language in Education | Other education |
| Religious Studies | Other humanities and arts |
| School Psychology | Psychology |
| Sociology | Sociology |
| Sociology of Education | Sociology |
| Spanish | Foreign languages and literature |
| Statistics | Mathematics and statistics |
| Stem Cell Biology and Regenerative Medicine | Biological and biomedical sciences |

**References**

[1] National Science Foundation. Survey of Earned Doctorates, http://www.nsf.gov/statistics/srvydoctorates/ (2018).
