# Supplementary material for: A latent class analysis approach to the identification of doctoral students at risk of attrition
Source: PLoS One. 2023 Jan 13;18(1):e0280325. doi: 10.1371/journal.pone.0280325 (PMC9838860; doi:10.1371/journal.pone.0280325)
Supplement: S6 Appendix — (DOCX) [file pone.0280325.s006.docx]

**S6 Appendix. Descriptive Information and Results for Individual Items.**

As noted in the main text, we examined individual items of scales when scale reliability was poor (i.e., Cronbach’s alpha <.75; see Table A for descriptive statistics). Specifically, we examined the individual items for strength of motivation, belonging uncertainty, and identity interference.

The individual strength of motivation items are as follows (note that we reverse-coded items 2 and 3; [1]:

1. Even if I could hardly maintain my social life, I would still continue graduate school.

2. It wouldn’t really bother me too much if I could no longer study my field.

3. I would quit studying if it became apparent that there were no jobs after graduation.

4. I would like to study my field, even if I have to spend a lot of time on topics that later turn out to be a waste of time.

Belonging uncertainty is comprised of three items [2]:

1. Sometimes I feel like I belong in grad school and sometimes I feel like I don't belong.
2. When something good happens, I feel like I really belong in grad school.
3. When something bad happens, I feel like maybe I don't belong in grad school.

Identity interference is comprised of four items (note that we reverse-coded items 2 and 3; [3]:

1. I feel that other [scientists/researchers] do not take me seriously because of my gender.
2. My gender makes me more capable as a [scientist/researcher].
3. I feel that because of my gender, it is easier for me to fit the definition of a [scientist/researcher].
4. It is hard to be my gender and a [scientist/researcher] in my field at the same time.

Results for individual items are similar to results for scales presented in main text (see Table B). Outcomes significantly vary by class for all individual items except for the third identity interference item—however, this is not surprising, as we indicated in the main text that this item was better removed from the identity interference scale given the results of a reliability analysis.

Note that for identity interference, we modified the phrasing of the items to be about gender in general rather than women specifically so that men and non-binary participants could complete the scale, and we changed the word “scientist” to “researcher” in the second cohort given not all participants were in a science-focused program of study.

**Table A. Descriptive Statistics on Outcomes of Individual Items from Scales with Low Reliability.**

| Variable | *n* | Mean (*SD)* | Range |
| --- | --- | --- | --- |
| **Academic Identity & Graduate School Attitudes** |  |  |  |
| Strength of Motivation #1 | 1081 | 5.03 (1.56) | 1 – 7 |
| Strength of Motivation #2 | 1081 | 5.36 (1.44) | 1 – 7 |
| Strength of Motivation #3 | 1081 | 4.66 (1.66) | 1 – 7 |
| Strength of Motivation #4 | 1080 | 4.45 (1.47) | 1 – 7 |
| **Interpersonal Relations & Perceived Fit** |  |  |  |
| Belonging Uncertainty #1 | 1080 | 4.49 (1.64) | 1 – 7 |
| Belonging uncertainty #2 | 1079 | 5.67 (1.10) | 1 – 7 |
| Belonging uncertainty #3 | 1078 | 3.63 (1.54) | 1 – 7 |
| **Social Identity Threat** |  |  |  |
| Identity interference item 1 | 1080 | 2.59 (1.68) | 1 – 7 |
| Identity interference item 2 | 1080 | 2.86 (1.65) | 1 – 7 |
| Identity interference item 3 | 1079 | 2.87 (1.65) | 1 – 7 |
| Identity interference item 4 | 1080 | 2.76 (1.77) | 1 – 7 |

**Table B. How Class Membership Predicts Descriptive Outcomes.**

| Variable | Lowest Threat  Mean (SE) | Nonchalant  Mean (SE) | Engaged/Worried  Mean (SE) | Highest Threat  Mean (SE) |
| --- | --- | --- | --- | --- |
| **Academic Identity & Graduate School Attitudes** |  |  |  |  |
| Strength of Motivation #1* | 5.365 (0.120) ^c, d^ | 4.650 (0.119) ^b, d^ | 5.299 (0.106) ^a, b^ | 4.820 (0.118) ^a, c^ |
| Strength of Motivation #2* | 5.887 (0.098) ^d, e^ | 4.930 (0.110) ^b, c, e^ | 5.919 (0.092) ^a, b^ | 4.587 (0.115) ^a, c, d^ |
| Strength of Motivation #3* | 5.178 (0.123) ^d, e^ | 4.386 (0.118) ^b, c, e^ | 4.972 (0.117) ^a, b^ | 4.027 (0.128) ^a, c, d^ |
| Strength of Motivation #4* | 4.777 (0.124) ^c, d^ | 4.196 (0.104) ^b, d^ | 4.765 (0.100) ^a, b^ | 4.019 (0.106) ^a, c^ |
| **Interpersonal Relations & Perceived Fit** |  |  |  |  |
| Belonging uncertainty #1* | 3.015 (0.123) ^b, d, e^ | 4.371 (0.109) ^a, c, e^ | 5.289 (0.106) ^a, b^ | 5.083 (0.096) ^c, d^ |
| Belonging uncertainty #2* | 5.744 (0.101) ^c, e^ | 5.589 (0.071) ^b, d^ | 6.075 (0.067) ^a, b, c^ | 5.097 (0.087) ^a, d, e^ |
| Belonging uncertainty #3* | 4.892 (0.105) ^b, d, e^ | 3.850 (0.101) ^a, c, e^ | 2.936 (0.107) ^a, b^ | 2.990 (0.099) ^c, d^ |
| **Social Identity Threat** |  |  |  |  |
| Identity interference item 1* | 1.950 (0.107) ^c, e^ | 1.762 (0.096) ^b, d^ | 3.658 (0.132) ^a, b, c^ | 2.866 (0.129) ^a, d, e^ |
| Identity interference item 2* | 2.522 (0.128) ^b, d^ | 2.614 (0.119) ^a, c^ | 3.248 (0.118) ^a, b^ | 3.004 (0.122) ^c, d^ |
| Identity interference item 3 | 2.724 (0.134) ^b^ | 2.845 (0.121) | 2.787 (0.114) ^a^ | 3.177 (0.126) ^a, b^ |
| Identity interference item 4* | 2.137 (0.120) ^c, e^ | 2.011 (0.104) ^b, d^ | 3.746 (0.139) ^a, b, c^ | 3.015 (0.132) ^a, d, e^ |

Asterisks beside outcome name indicate overall Chi-square test was significant at *p* < .05. Matching letters denote significant pairwise differences at *p* < .05.

**References**

[1] Nieuwhof MGH, Cate OT ten, Oosterveld P, Soethout MBM. Measuring strength of motivation for medical school. *Med Educ Online* 2004; 9: 4355.

[2] Walton GM, Cohen GL. A question of belonging: Race, social fit, and achievement. *J Pers Soc Psychol* 2007; 92: 82–96.

[3] Settles IH. When multiple identities interfere: The role of identity centrality. *Pers Soc Psychol Bull* 2004; 30: 487–500.
