# Supplementary material for: A latent class analysis approach to the identification of doctoral students at risk of attrition
Source: PLoS One. 2023 Jan 13;18(1):e0280325. doi: 10.1371/journal.pone.0280325 (PMC9838860; doi:10.1371/journal.pone.0280325)
Supplement: S7 Appendix — (DOCX) [file pone.0280325.s007.docx]

**S7 Appendix. Trichotimization Details.**

**Table A. Trichotimization Cutoffs for Indicators.**

| Indicator | Low (*n*) | Medium (*n*) | High (*n*) | Range |
| --- | --- | --- | --- | --- |
| Need fulfillment composite | ≤ -0.5 (283) | -0.5 < x < 0.5 (489) | ≥ 0.5 (308) | -3.66 – 1.96 |
| Gender threat | ≤ 3.49 (758) | 3.49 < x < 4.49 (171) | ≥ 4.49 (151) | 1 – 7 |
| Academic and social concerns | ≤ 3.49 (270) | 3.49 < x < 4.49 (295) | ≥ 4.49 (515) | 1 – 7 |
| Impostor syndrome | ≤ 2.49 (267) | 2.49 < x < 3.49 (394) | ≥ 3.49 (419) | 1 – 5 |
| Grit | ≤ 2.49 (69) | 2.49 < x < 3.49 (433) | ≥ 3.49 (579) | 1.12 – 5 |
| Distress | ≤ 7 (627) | 7 < x < 13 (374) | ≥ 13 (78) | 0 – 24 |
| Interest | ≤ 5 (116) | 5 < x < 6 (392) | ≥ 6 (573) | 2 – 7 |
| Researcher identification | ≤ 5 (393) | 5 < x < 6 (506) | ≥ 6 (182) | 1.6 – 7 |
