# Supplementary material for: A latent class analysis approach to the identification of doctoral students at risk of attrition
Source: PLoS One. 2023 Jan 13;18(1):e0280325. doi: 10.1371/journal.pone.0280325 (PMC9838860; doi:10.1371/journal.pone.0280325)
Supplement: S8 Appendix — (DOCX) [file pone.0280325.s008.docx]

**S8 Appendix. Indicators Considered for LCA.**

As noted in the main text, we considered multiple variables as indicators for a latent class analysis model capturing incoming Ph.D. students’ psychological threat experiences. In various combinations, we tried all of the variables in List A as indicators of a latent class analysis. Variables that did not distinguish between classes well were eliminated.

**List A. Indicators Considered for LCA.**

- Stereotype threat
- Academic and social concerns
- Academic belonging
- Impostor syndrome
- Grit
- Distress
- Stigma consciousness
- Identity interference
- Need for autonomy
- Need for connection
- Need for competence
- Need for autonomy/connection/competence combined
- Graduate school self-efficacy
- Person-environment match
- Researcher identification
- Interest in research item 1 (“I like doing research”)
- Interest in research item 2 (“I am interested in my research topic”)
- Interest in field
- Strength of motivation
- Stress mindset
- Need for structure item 3 (“I don't like situations that are uncertain”)
- Communal affordances item 2 (“How much does a career in your field involve helping society?”)
- Gender identification
- Stereotype perception (from endorsement scale) gender/society item
- Stereotype perception (from endorsement scale) race/society item
- Everyday discrimination
- Growth mindset

*Note.* Stimuli and sources for these measures are included among other baseline materials, available at https://osf.io/updy9/.
