# Supplementary material for: A latent class analysis approach to the identification of doctoral students at risk of attrition
Source: PLoS One. 2023 Jan 13;18(1):e0280325. doi: 10.1371/journal.pone.0280325 (PMC9838860; doi:10.1371/journal.pone.0280325)
Supplement: S9 Appendix — (DOCX) [file pone.0280325.s009.docx]

**S9 Appendix. Composite Indicator Variables.**

The need fulfillment variable was comprised of academic belonging, graduate school self-efficacy, and psychological need satisfaction. These constructs loaded onto the same factor in a principal components analysis (PCA), and conceptually overlap in that belonging and self-efficacy are components of need satisfaction (e.g., [1]).* To create the need fulfillment composite, we standardized each of the three aforementioned measures and calculated an average (α = .76) and then trichotomized the composite variable. Because we transformed the data, we chose cutoffs based on the distribution of responses (i.e., the standard deviations and percentage of people in each cell; see S7 Appendix), rather than based on scale meaning.

The gender threat composite was based on the gender-based stereotype threat scale and two items from the identity interference scale, which loaded together in a PCA. The two identity interference items were: “I feel that other [scientists/researchers] do not take me seriously because of my gender” and “It is hard to be my gender and a [scientist/researcher] in my field at the same time.” The resulting composite variable, hereafter called gender threat, had good reliability (α = .85). We then trichotomized the gender threat composite based on scale scores and anchors because the items were all measured using the same scale (see S7 Appendix).

[**Note.* We also tested the unrestricted 4-class LCA for the ultimately selected model using the individual need fulfillment variables (i.e., academic belonging, self-efficacy, and psychological need satisfaction) and found a substantively similar solution, although the percentage of people in each class differed somewhat. This model had high similarity in the item distributions by class for the three variables that we ultimately collapsed into a composite, suggesting the value in the more parsimonious model using the composite.]

**References**

[1] Kassis W, Graf U, Keller R, Ding K, Rohlfs C. The role of received social support and self-efficacy for the satisfaction of basic psychological needs in teacher education. *Eur J Teach Educ* 2019; 42: 391–409.
