# Supplementary material for: A latent class analysis approach to the identification of doctoral students at risk of attrition
Source: PLoS One. 2023 Jan 13;18(1):e0280325. doi: 10.1371/journal.pone.0280325 (PMC9838860; doi:10.1371/journal.pone.0280325)
Supplement: S10 Appendix — (DOCX) [file pone.0280325.s010.docx]

**S10 Appendix. Visual Presentation of Characteristic Means by Class.**

In this section, we present in figures the estimated means of the proximal characteristics by class to facilitate interpretation (see Figs A-E). Equivalent information is presented in Table 7 of the main text. Note that higher numbers indicate more of the construct except for academic career preference, which was scaled from -5 (*strongly prefer non-academic*) to 5 (*strongly prefer academic*).

**Figure A. Academic Preparation and Context Characteristics by Class.**

**
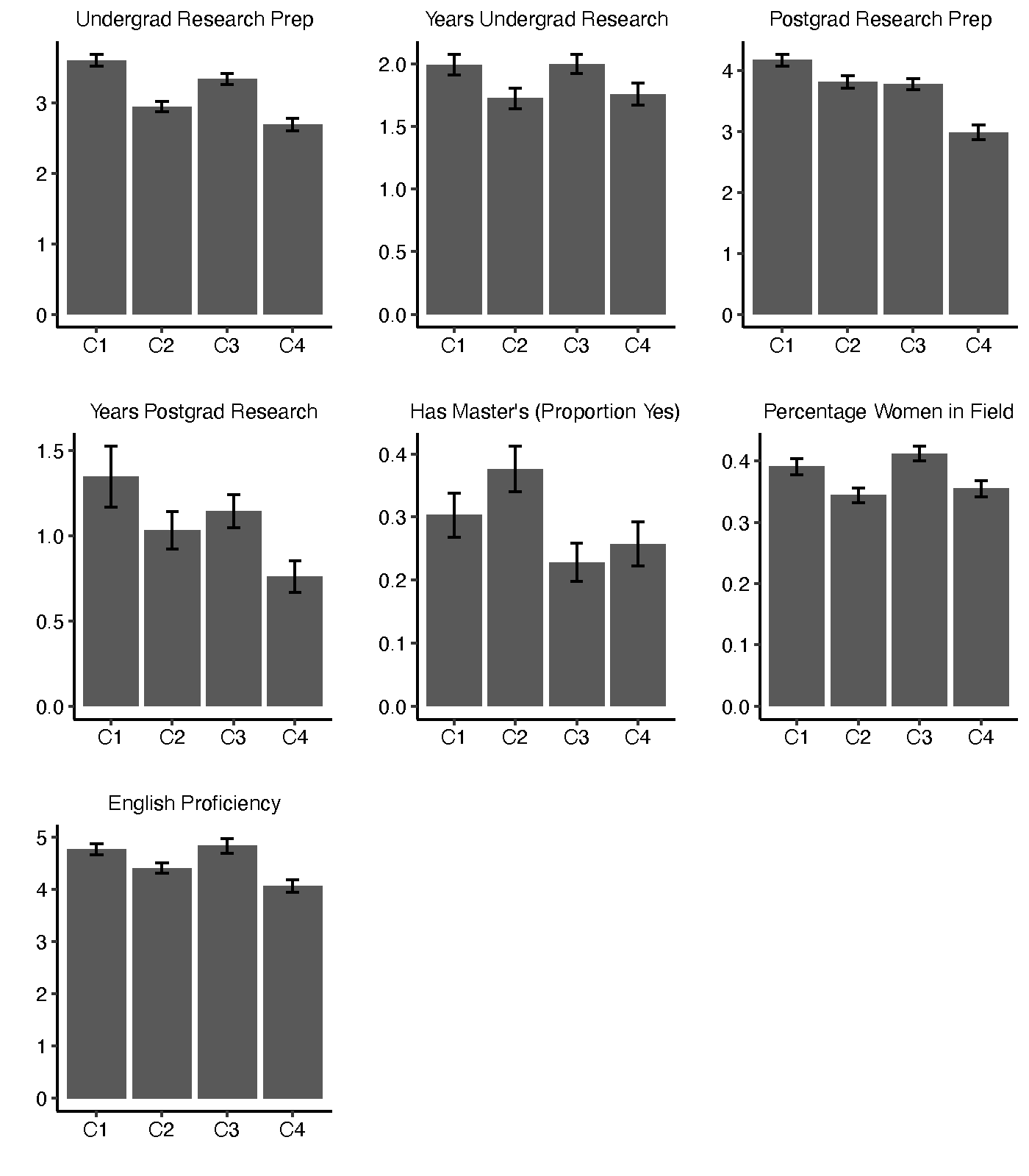
**

C1: Lowest Threat, C2: Nonchalant, C3: Engaged/Worried, C4: Highest Threat. Error bars represent standard errors of the mean as estimated by the LCA analyses using the BCH procedure.

**Figure B. Academic Identity and Graduate School Attitude Characteristics by Class.**

*
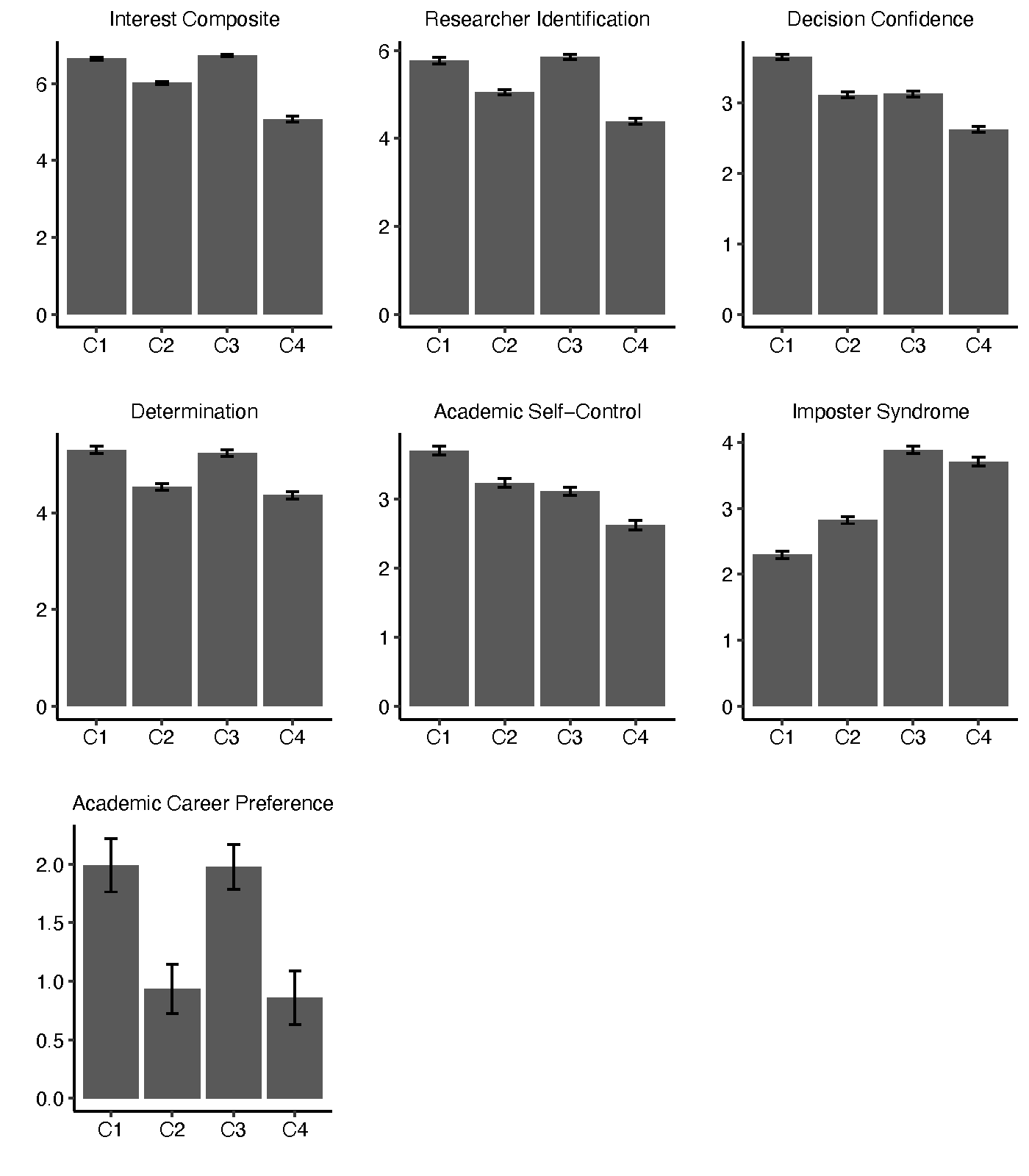
*

C1: Lowest Threat, C2: Nonchalant, C3: Engaged/Worried, C4: Highest Threat. Error bars represent standard errors of the mean as estimated by the LCA analyses using the BCH procedure.

**Figure C. Interpersonal Relations and Perceived Fit Characteristics by Class.**

*
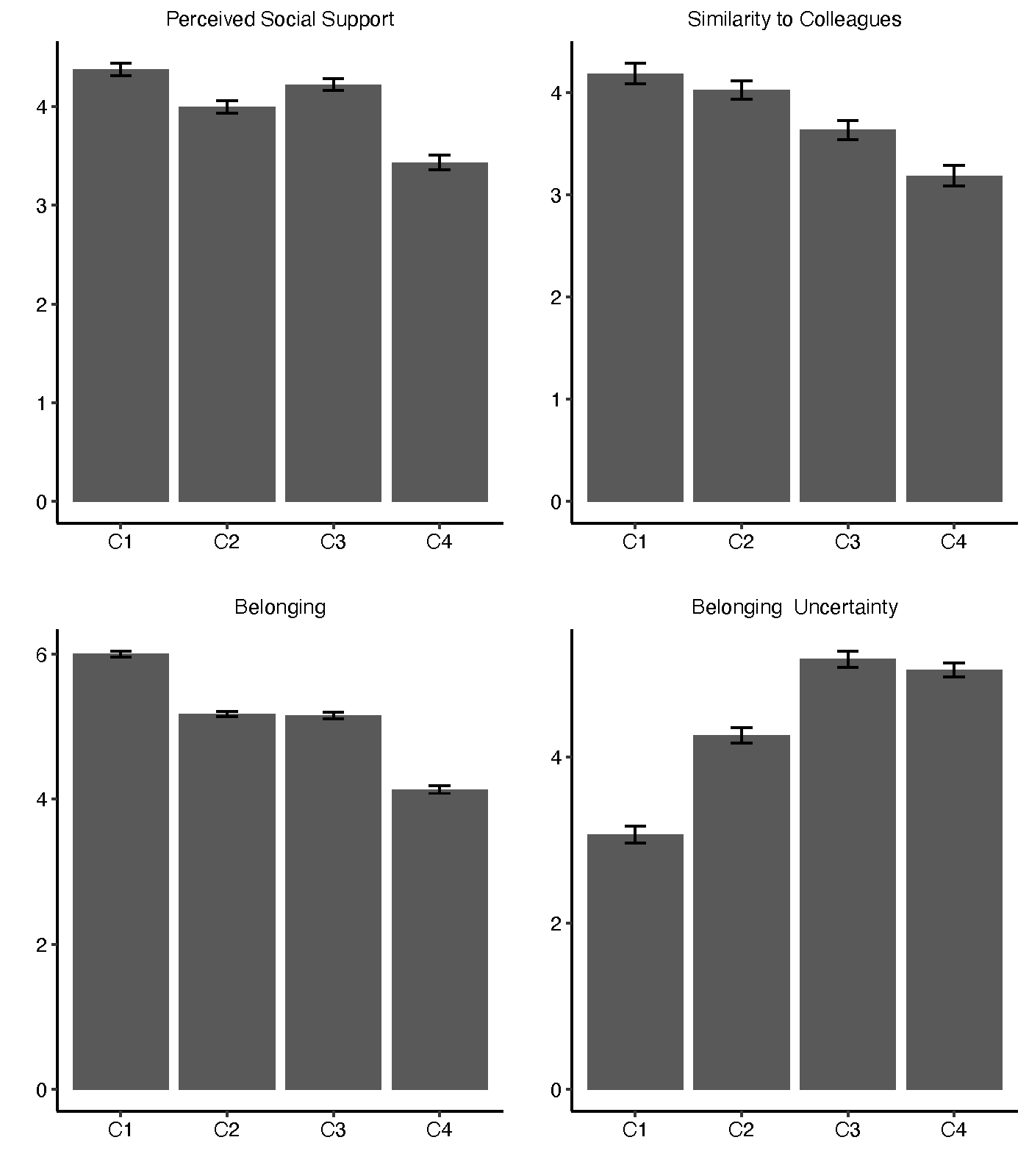
*

C1: Lowest Threat, C2: Nonchalant, C3: Engaged/Worried, C4: Highest Threat. Error bars represent standard errors of the mean as estimated by the LCA analyses using the BCH procedure.

**Figure D. Personality and Self-Evaluations by Class.**


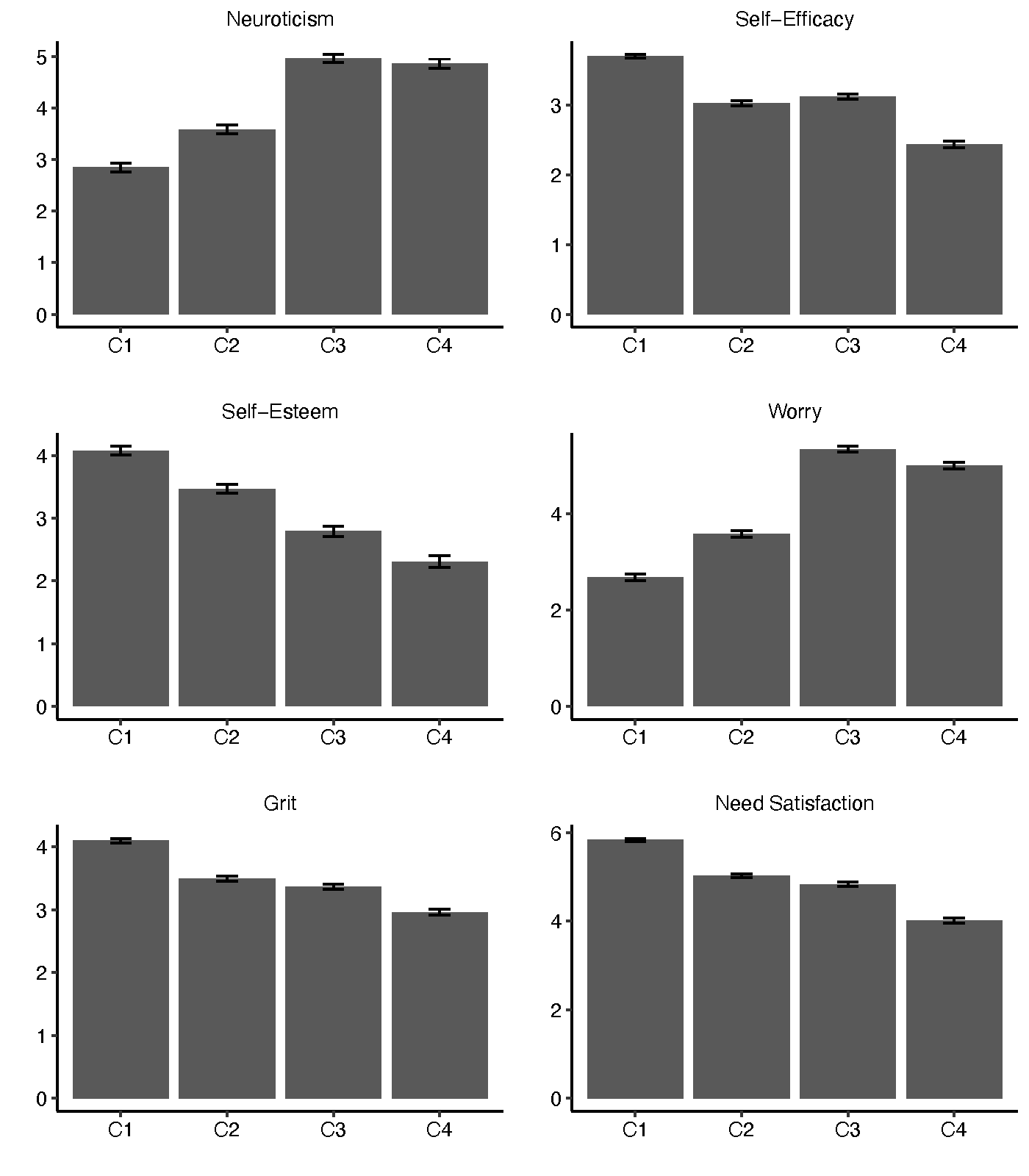


C1: Lowest Threat, C2: Nonchalant, C3: Engaged/Worried, C4: Highest Threat. Error bars represent standard errors of the mean as estimated by the LCA analyses using the BCH procedure.

**Figure E. Mental Health and Social Identity Threat by Class.**

**
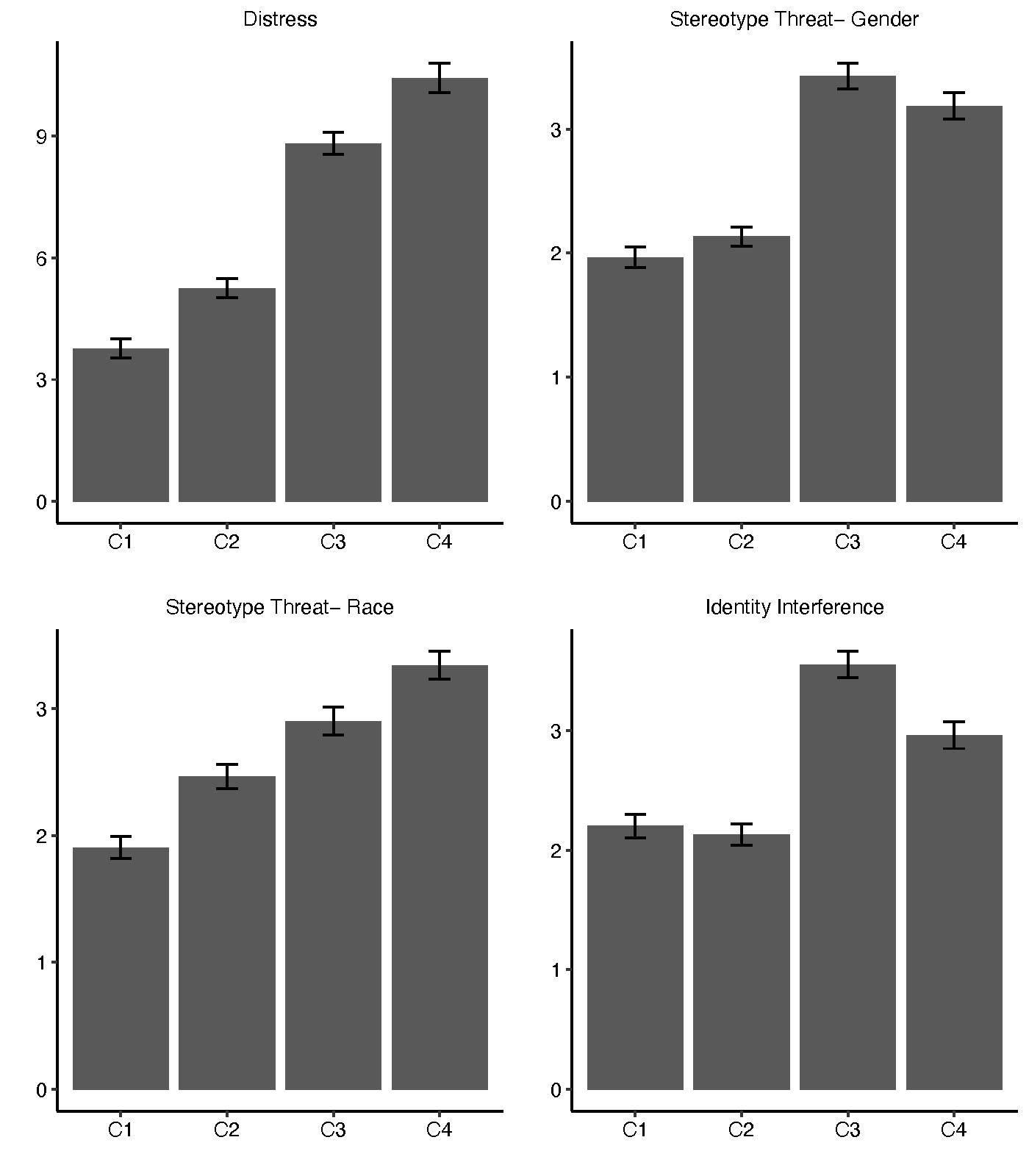
**

C1: Lowest Threat, C2: Nonchalant, C3: Engaged/Worried, C4: Highest Threat. Error bars represent standard errors of the mean as estimated by the LCA analyses using the BCH procedure.
