# Supplementary material for: A latent class analysis approach to the identification of doctoral students at risk of attrition
Source: PLoS One. 2023 Jan 13;18(1):e0280325. doi: 10.1371/journal.pone.0280325 (PMC9838860; doi:10.1371/journal.pone.0280325)
Supplement: S11 Appendix — (DOCX) [file pone.0280325.s011.docx]

**S11 Appendix. LCA Subsample (Robustness) Analyses.**

We conducted robustness analyses by campus and cohort. As evidenced in Figs A-D, highly similar 4-class models emerged for each campus and cohort subsample. When examining campus subsamples, we grouped Columbia and Stanford together because we recruited smaller samples at these universities than at Penn State and were concerned about the sample size being too small for LCA if we analyzed each university separately. Given that highly similar classes emerged regardless of subsample used, we contend that these results highlight the robustness of our model.

**Figure A. Item Distribution by Class for Penn State Subsample Across Both Cohorts.**


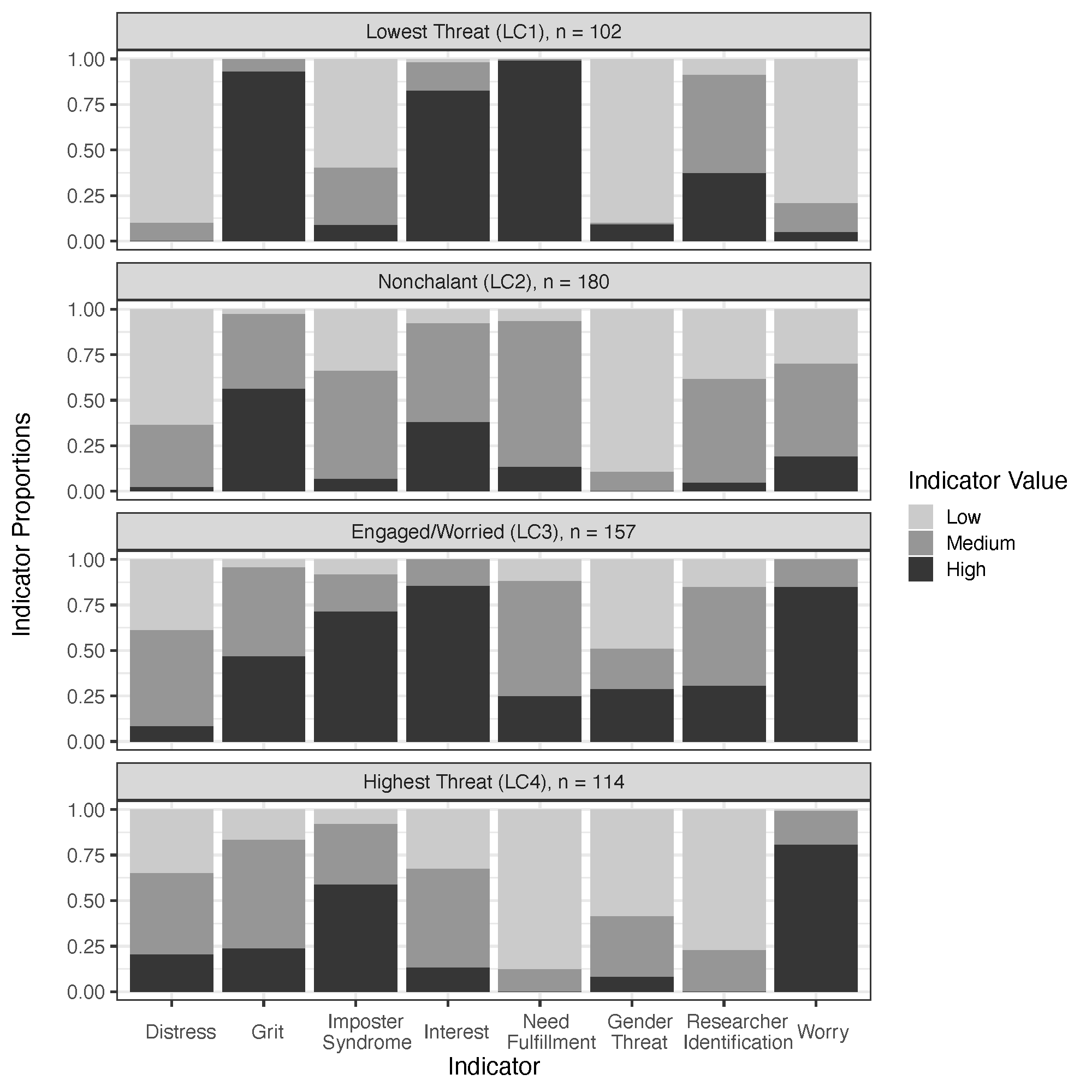


LC = Latent Class. “Worry” refers to Academic and Social Concerns.

**Figure B. Item Distribution by Class for Columbia and Stanford Subsample Across Both Cohorts.**


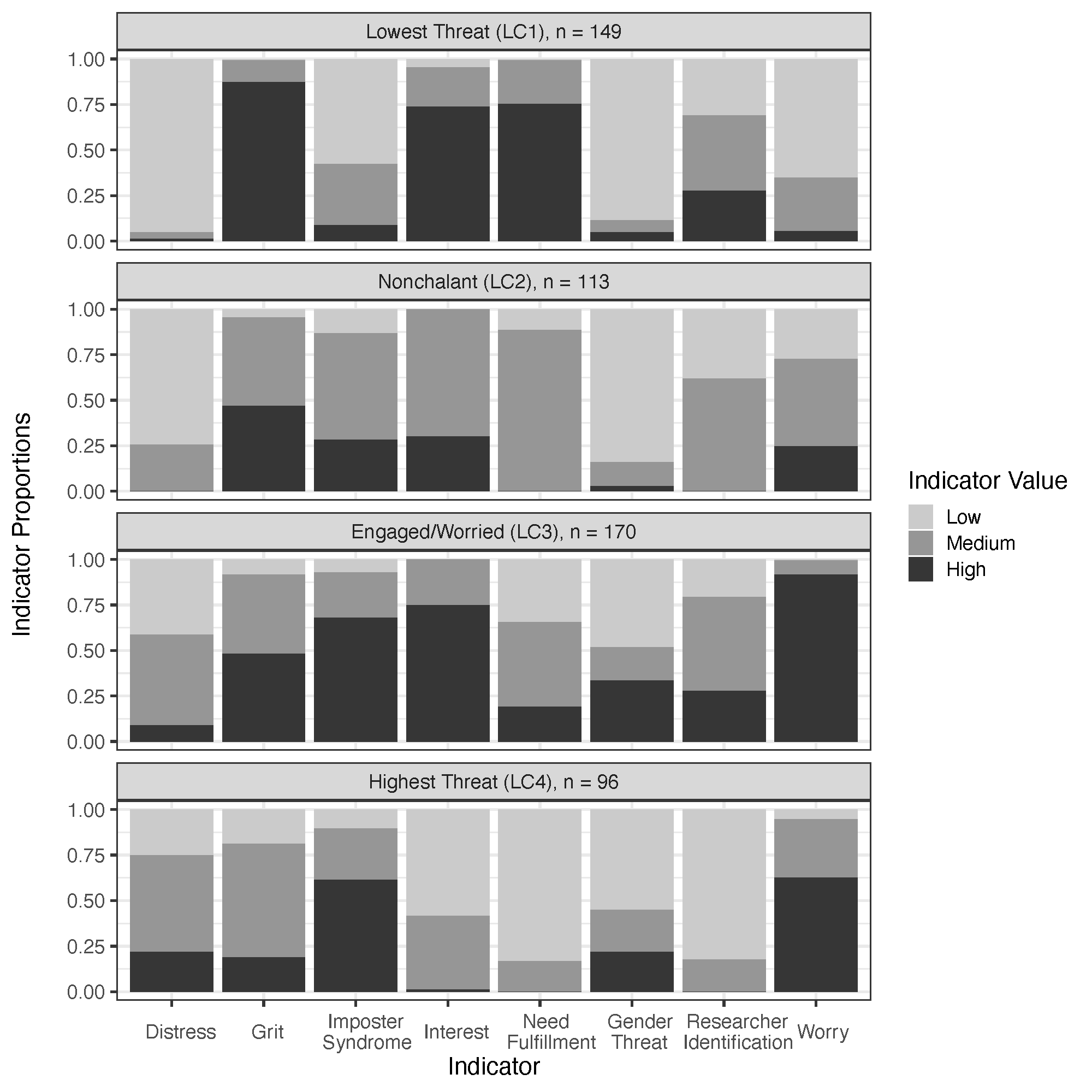


LC = Latent Class. “Worry” refers to Academic and Social Concerns.

**Figure C. Item Distribution by Class for First Cohort Subsample Across Universities.**


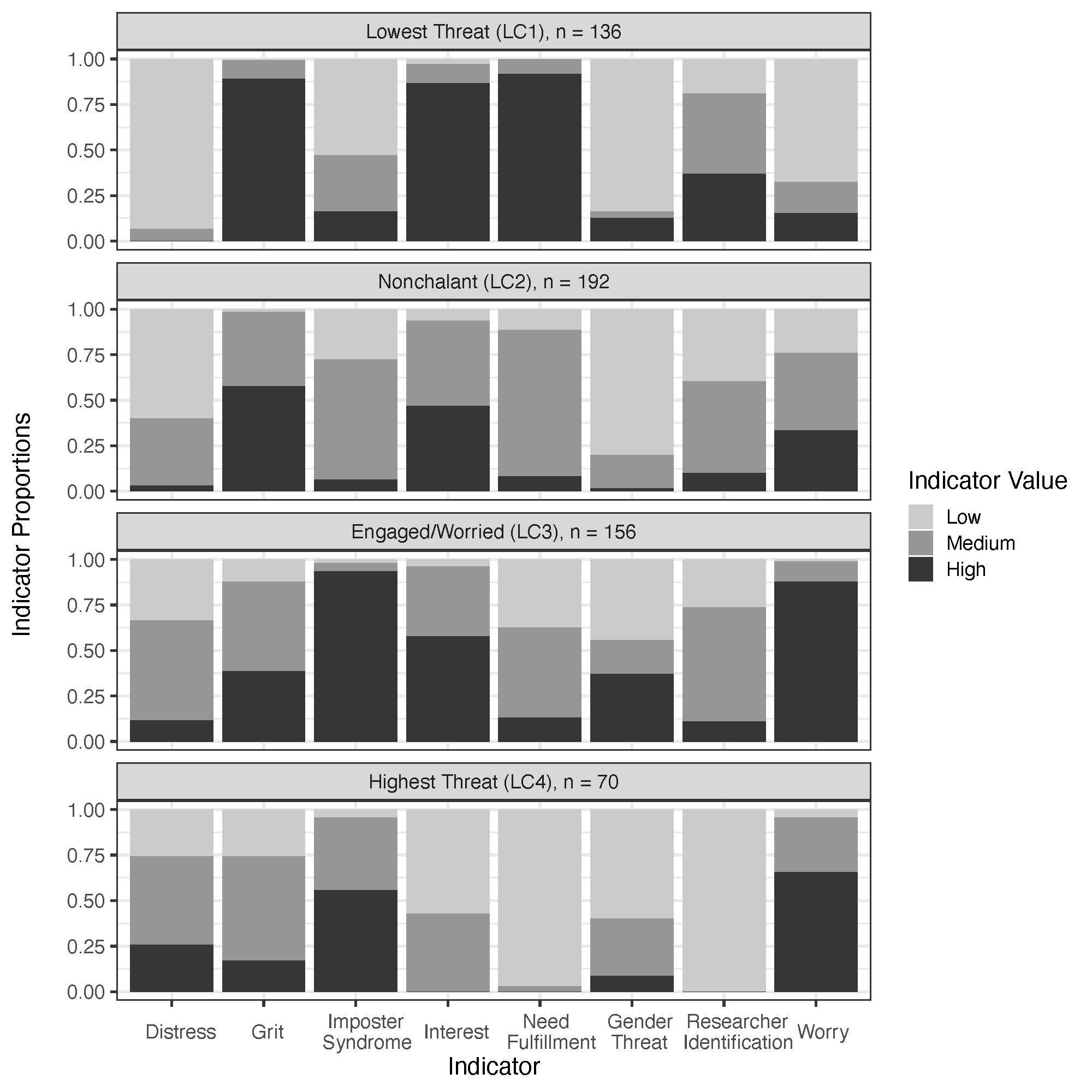


LC = Latent Class. “Worry” refers to Academic and Social Concerns.

**Figure D. Item Distribution by Class for Second Cohort Subsample Across Universities.**


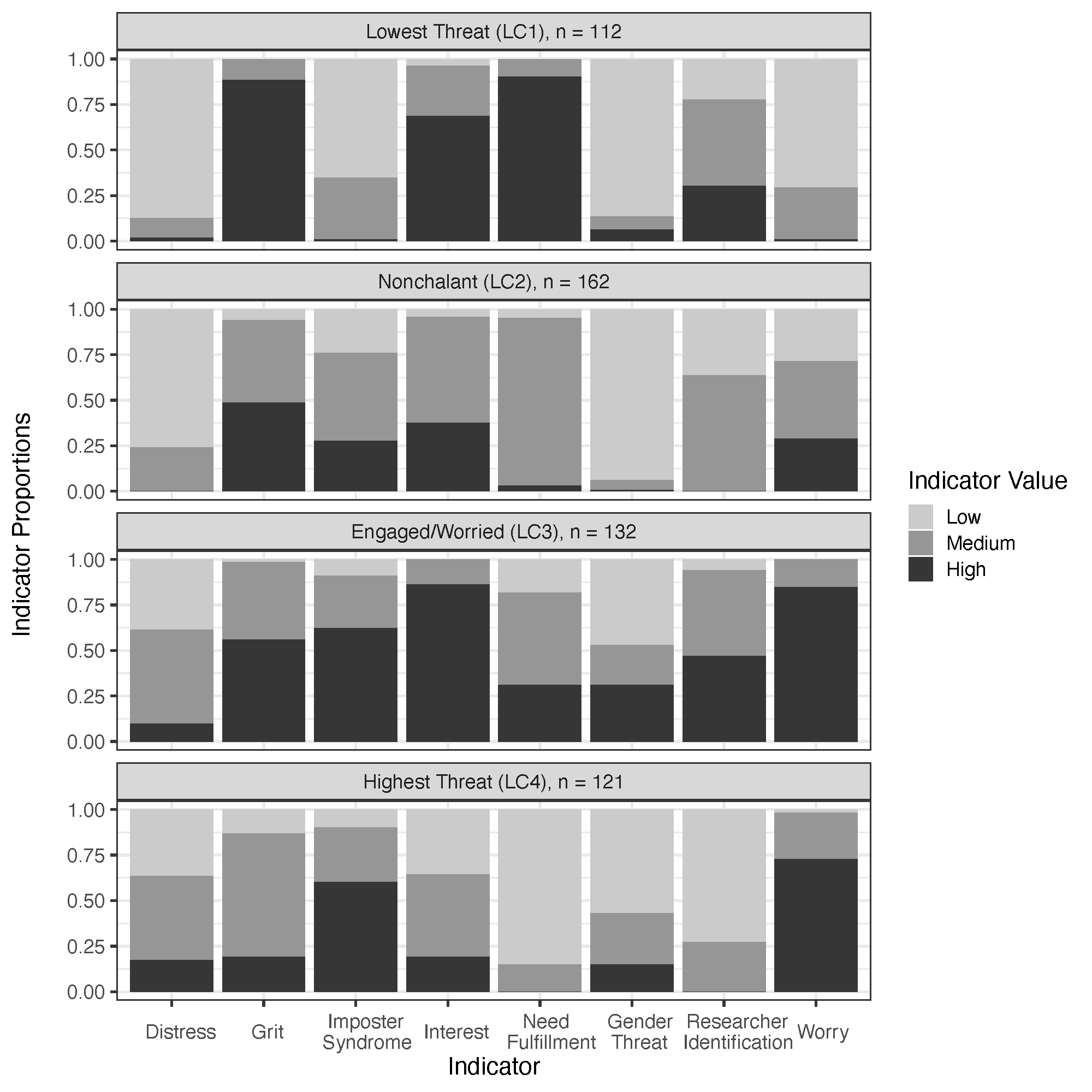


LC = Latent Class. “Worry” refers to Academic and Social Concerns.
